# The CRY1-HsF predicted interaction interface serves as a molecular platform for bioengineering or selecting modulating mutants

**DOI:** 10.3389/fpls.2025.1712571

**Published:** 2025-12-17

**Authors:** Souleïmen Jmii, William Bouard, Gabriel Marcotte, Julien Plamondon, Laurent Cappadocia

**Affiliations:** 1 Department of Chemistry, Université du Québec à Montréal, Montréal, QC, Canada; 2 Centre Sève, Department of Biology, Université de Sherbrooke, Sherbrooke, QC, Canada; 3 Quebec Network for Research on Protein Function, Engineering and Applications (PROTEO), Québec, QC, Canada; 4 Department of Biological Sciences, Université du Québec à Montréal, Montréal, QC, Canada

**Keywords:** Arabidopsis, bioengineering, structure prediction, protein-protein interactions, CRY1, HsF, mutants

## Abstract

**Introduction:**

High-temperature stress imposes an energetic cost on plant growth and negatively impacts agricultural productivity. This stress rapidly triggers the activation of Heat shock Factor (HsF) proteins, a family of transcription factors that maintain proteostasis. The cryptochrome CRY1 can physically interact with HsFA1d proteins to facilitate nucleus translocation and the regulation of genes that contributes to stress tolerance.

**Methods:**

We combined structural predictions with experimental testing using yeast-two-hybrid and bimolecular fluorescence complementation assays.

**Results:**

We confirm that CRY1 PHR domain interacts extensively with multiple HsF proteins through their HR-A region of the conserved oligomerization HR-A/B domain interface. This interaction partially relies on salt bridges provided by the N- and C-terminus of HR-A region and a conserved interface centered around W352 of CRY1. HsFA3 shows the strongest affinity to CRY1 in yeast-two-hybrid assays notably thanks to a glutamate residue that interacts with R211 and R435 of CRY1. Mutating equivalent residue positions within HsFA1e or HsFC1 to a glutamate increased their interaction to CRY1.

**Discussion:**

Overall, our analysis allowed the identification of mutant candidate that could be used in selection or bioengineering endeavors to improve thermal stress tolerance.

## 1 Introduction

High-temperature stress limits plant growth and directly suppresses agricultural productivity (Yu et al., 2024). In response to a high temperature stress, plants activate a conserved Heat Shock Response (HSR), characterized by light-induced thermotolerance (Dündar et al., 2025). The initiation of the HSR is tightly linked to environmental temperature sensing, a process in part mediated by blue-light photoreceptors, cryptochromes 1 and 2 (CRY1 and CRY2). CRY1-mediated signaling enhances the expression of Heat shock Factors (HsFs), a diverse family of 21 transcriptional regulators organized into three distinct classes (15 activating Class A; 5 repressive Class B and 1 regulatory Class C) that maintain proteostasis under stress (Nover et al., 2001; Gao et al., 2023). HsFs proteins form homo and heterotrimers, reinforcing transcriptional network complexity and enabling efficient signal amplification (Yoshida et al., 2011; Friedrich et al., 2021). Following heat stress, a hierarchical activation cascade occurs between HsFs proteins, from HsFA1d to Heat Shock Elements (HSE) on the chromatin, and drive the expression of specific heat stress tolerance genes (Ohama et al., 2016). CRY1 and CRY2 interact physically with HsFA1s subfamily members, promoting nuclear translocation via recruitment of the importin α1 (IMPα1) nuclear import receptor (Gao et al., 2023). CRY1 and CRY2 possess an N-terminal photolyase homology region (PHR), which binds the flavin adenine dinucleotide (FAD) cofactor upon for light detection. This domain allows the formation of stable oligomers and masking certain interacting surfaces, including tryptophan 352 which participate to dimerization (Shao et al., 2020). The CRY1 PHR domain interacts physically with a HsFA1d fragment composed of residues 1–220 aa that lacks the C-terminal transactivation or repression domain but otherwise comprises an N-terminal DNA-binding domain (DBD) that recognize HSE on the chromatin, a disordered linker, and the HR-A/B domain formed of two Heptad Repeat, HR-A and HR-B regions that mediate oligomerization (Nover et al., 2001; Gao et al., 2023). As for the CRY1 protein, it possesses a C-terminal regulatory region, which is intrinsically disordered and essential for photoactivation triggering monomerization, enabling interaction with downstream partners and facilitating signal transduction (Shao et al., 2020). Chromatin immunoprecipitation sequencing (ChIP-seq) shows that CRY1 and HsFA1d co-occupy a broad set of genomic loci, with RNA-seq identifying 42 co-regulated genes, predominantly encoding HSPs and stress-responsive factors. These genes remain transcriptionally silent in *cry1* and *hsfa1a/b/d/e* quadruple mutants, even under light or heat stress, indicating a critical regulatory role for CRY1 in HsFA1-mediated transcription. ChIP-qPCR confirms reduced expression of key HSR genes (e.g., *HSFB1*, *HSFB2b*) in the absence of CRY1, supporting its function as a stabilizing factor in the transcriptional complex. Loss-of-function mutants *cry1–304* and *cry1-349*, which lack functional CRY1, fail to induce HSP expression upon light exposure and are unable to acquire light-induced thermotolerance, underscoring the essential role of CRY1. In contrast, the *cry2–1* mutant exhibit partial reduction in thermotolerance, suggesting a compensatory or overlapping function for CRY2. Meanwhile, HsFA1d induces the expression of HsFA3 and its overexpression confers heat stress and drought stress tolerance characterized by reduced wilting, lower levels of reactive oxygen species (ROS), and enhanced accumulation of proline, alongside elevated activity of ROS-scavenging enzymes: CAT, APX, SOD (Li et al., 2013; Song et al., 2016; Zhu et al., 2020; Ren et al., 2024). Overall, the CRY1-HsFA1s complex thus represents a key early sensor-effector module in the heat stress response. Given the high sequence conservation among HsFs, their functional redundancy, and the important role of HsFA3 in thermotolerance we propose that CRY1 may serve as a scaffold for the stabilization and assembly of multi-protein transcriptional complexes. Guided by AlphaFold predictions and structural homology modeling, we performed a detailed analysis of the interaction interface between CRY1 and HsFs members using yeast-two-hybrid (Y2H) and bimolecular complementation fluorescence assays (BiFC). The identification of key interacting residues provides a foundation for the rational design of gain-of-function mutants inspired by natural sequence variation.

## 2 Materials and methods

### 2.1 Sequence alignments of Arabidopsis HsFs and plant homologues of HsFA3

The 21 HsFs proteins sequences from *Arabidopsis thaliana* and the 25 HsFs proteins sequences from *Oryzia sativa* were obtained from UniProtKB, accession numbers are listed respectively in **Supplementary Tables 1** and **2**. Plant’s HsFA3 proteins amino acid sequences were obtained from HMMER v3.4 (Finn et al., 2011) and their accession numbers are listed in **Supplementary Table 3**. Sequences were aligned using NCBI BLAST (Altschul and Lipman, 1990) and alignment were visualized using Espript software v.3 (Robert and Gouet, 2014) or Web Logo 3 (Crooks et al., 2004).

### 2.2 Structural prediction of the CRY1-HsFA3 complex

The structure of CRY1-HsFA3 was predicted by homology modeling using SWISS model (Waterhouse et al., 2018) and the AlphaFold prediction of CRY1-HsFA1d as a template. The structure of Arabidopsis CRY1 and HsFA1s complexes were obtained through AlphaFold v.3 (using accession number listed in **Supplementary Table 3**). The visualization software PyMOL (The PyMOL Molecular Graphics System, Version 2.5.2 Schrodinger, LLC.) was used to analyze the structure.

### 2.3 Molecular cloning, mutagenesis and proteins stability

Vectors coding for AtCRY1 (#G12079) and HsFs proteins: HsFA1a (#DKLAT4G17750), HsFA1b (amplified on Arabidopsis cDNA), HsFA1d (#PADLOX-TF-13-E02), HsFA1e (#U85727, HsFA3 (#PADLOX-TF-11-A02), HsFA4C (#G67044), HsFA6a (#PADLOX-TF-08-F01), HsFA6b (#G10337), HsFA7a (#U23517), HsFA9 (#U18576), HsFB2b (#U16604), HsFB3 (#U86661), HsFC1 (#U14335) were obtained from the Arabidopsis biological resource center (ABRC). Polymerase chain reaction (PCR) was used to amplify the full-length CRY1 sequence (residues 1–682), the CRY1 PHR domain (residues 1–508 aa), and the full-length HsFs sequences, which were subsequently cloned into yeast and plant expression vectors using GIBSON assembly. Site-directed mutagenesis was performed using Gibson assembly with specific oligonucleotides (**Supplementary Table 4**) to introduce substitutions or deletions, generating CRY1 and HsFA3 mutants. All plasmids were confirmed by Sanger sequencing (Genome Québec) or Nanopore sequencing (Plasmidsaurus). Stability of point mutants was evaluated using mCSM (Pires et al., 2014), which estimates the thermodynamic impact of single amino acid substitutions on the protein structural stability.

### 2.4 Yeast 2-hybrid assay and β -galactosidase quantification

Sequences were cloned by GIBSON assembly into pGADT7 and pGBKT7 yeast plasmids. Empty GAL4 bait and prey vector were used as negative controls. Multiple combinations of prey (AD) and bait (BD) were tested using the following set of plasmids: (AD) CRY1^WT^, CRY1^PHR WT^, CRY1^PHR W352A^, CRY1^PHR K332A^, CRY1^PHR K332E^, CRY1^PHR R335A^, CRY1^PHR R335E^, CRY1^PHR K332E R335E^, CRY1^PHR R211A^, CRY1^PHR R211E^, CRY1^PHR R435A^, CRY1^PHR R435E^, CRY1^PHR R211E R435E^; (BD): HsFA1a^WT^, HsFA1b^WT^, HsFA1d^WT^, HsFA1e^WT^, HsFA1e^R126E^, HsFA3^WT^, HsFA4C^WT^, HsFA6a^WT^, HsFA6b^WT^, HsFA7^WT^, HsFA9^WT^, HsFB2b^WT^, HsFB2b^K249E^, HsFB3^WT^, HsFC1^WT^, HsFC1^R129E^, HsFA3^E173A^, HsFA3^E173R^, HsFA3^K174A^, HsFA3^K177A^, HsFA3^E178A^, HsFA3^K174A K177A^, HsFA3^K174A K177A E178A^. Bait and prey constructs were co-transformed into yeast strain Y187 (TaKaRa) and selected on synthetic lacking leucine and tryptophan dropout medium (SD/-LW) for 3 to 5 days at 30°C. Four random colonies from each transformation were re-selected on (SD/-LW) for 3 days at 30°C. At least three colonies for each transformation pair were selected and resuspended to grow overnight in liquid SD/-LW. Cultures were diluted until 0.05 of optical density (OD)_600_ in liquid rich YPD and grown further until the optical density reached a 0.5 – 0.8 range. The culture was then pelleted and resuspended in 300 µL of lysis buffer containing 100 mM of 4-(2-hydroxyethyl)-1-piperazineethanesulfonic acid (HEPES), 0.150 mM of NaCl, 5 mM of L-aspartate, 1% (w/v) BSA and 0.05% (v/v) Tween-20, pH adjusted to 7.25– 7.30). Cells were disrupted by repetitively freezing the culture in liquid nitrogen, followed by rapid thawing in a 37°C water bath. 700µL of reaction buffer (2.23 mM chlorophenol red β-D-galactopyranoside (CPRG) Sigma Aldrich) was then added to start the reaction. The reaction was stopped when the color of the sample turned orange/red by adding 0.5 ml of 3 mM ZnCl_2_. Cell debris were removed by spinning and the OD_578_ of the supernatant was measured using Thermo Scientific NanoDrop. β-galactosidase activity (Miller units) was calculated using the following equation:


$$activity=\frac{1000\;\times \;OD\;578}{t\;\times \;V\;\times \;OD\;600}$$

where y is the β-galactosidase unit; t is the elapsed time (in minutes) of incubation; V is 0.1 x concentration factor (in this case V = 0.5). An interaction was deemed ‘not detected’ (ND) if the OD_578_ was<0.01 after 3 h of color development.

### 2.5 Plant material and growth conditions

*Nicotiana benthamiana* (PI 555478 USDA) grown in a mixture of black earth, perlite and peat moss (2:1:1, v:v:v) in E15 Conviron growth cabinet at 22°C and 70% relative humidity, under a photon flux density of 100µmol m-2 s-1 (fluorescent and incandescent lighting) and a 16h/8h (day/night) photoperiod. Plants were watered with 20:20:20 N:P:K at a concentration of 0.5g.L^-1^, and infiltration was performed on 5 weeks old leaves.

### 2.6 Bimolecular complementation by fluorescence assay

To assess CRY1 interaction with HsFs proteins, we amplified CRY1 and HsFs coding sequences and inserted these sequences between the XbaI and NcoI restriction enzyme of a modified pAVA321 vector (Von Arnim et al., 1998) generating 35S:nYFP (1-156) or pAVA 35S:cYFP (155-240) vectors. This generates pAVA 35S:CRY1-nYFP and pAVA 35S:HsFs-cYFP where eYFP N-terminus or C-terminus are respectively separated from CRY1 and HsFs by a 4xGlycine linker. CRY1^WT^, CRY1^PHR^, HsFA1d^WT^, HsFA1d^Δ(160-220)^, HsFA1e^WT^, HsFA1e^R126E^, HsFA3^WT^, HsFA3^Δ(168-228)^, HsFB2b^WT^, HsFB2b^K249E^, HsFC1^WT^, HsFC1^R129E^ sequences were cloned into pAVA modified vectors using GIBSON assembly to generate pAVA 35S:CRY1^WT^-nYFP, pAVA 35S:CRY1^PHR^-nYFP, pAVA 35S:HsFA3^WT^-cYFP and pAVA 35S:HsFA3^Δ(168-228)^ -cYFP using the 5’ XbaI site. The resulting cassettes were excised and inserted in pPZP vector in the SmaI and KpnI sites to generate pPZP 2X35S:CRY1^WT^-nYFP, pPZP 2X35S:CRY1^PHR^-nYFP, pPZP 2X35S:HsFA1e^WT^-cYFP, pPZP 2X35S:HsFA1e^R126E^-cYFP, pPZP 2X35S:HsFA3d^WT^-cYFP and pPZP 2X35S:HsFA3^Δ(168-228)^-cYFP, pPZP 2X35S:HsFB2b^WT^-cYFP, pPZP 2X35S:HsFB2b^K249E^-cYFP, pPZP 2X35S:HsFC1^WT^-cYFP, pPZP 2X35S:HsFC1^R129E^-cYFP. We used pPZP 2X35S:ECoil-cYFP and pPZP 2X35S:RCoil-nYFP as a positive control (Doh et al., 2018), whereas pPZP 2X35S:mCherry signal serves as a marker for transient expression and tissue localization in agro-infiltrated leaves. It also serves as an internal control to normalize fluorescence between the different tested combinations. All pPZP plasmids mentioned previously were transformed into *Agrobacterium tumefaciens* EHA105 to generate individual strains. Agroinfiltration of *Nicotiana benthamiana* leaves were performed as described (Bouard et al., 2024). Two days after infiltration, fluorescence excitation/emission wavelengths of 488 nm/525 nm for eYFP and 561 nm/595 nm for mCherry were used on a Nikon A1+ confocal laser scanning microscope. Images were analyzed using Fiji software (Schindelin et al., 2012).

## 3 Results

### 3.1 Similarities in HsFs class A and C structure allow interaction with CRY1

**Figure 1:**
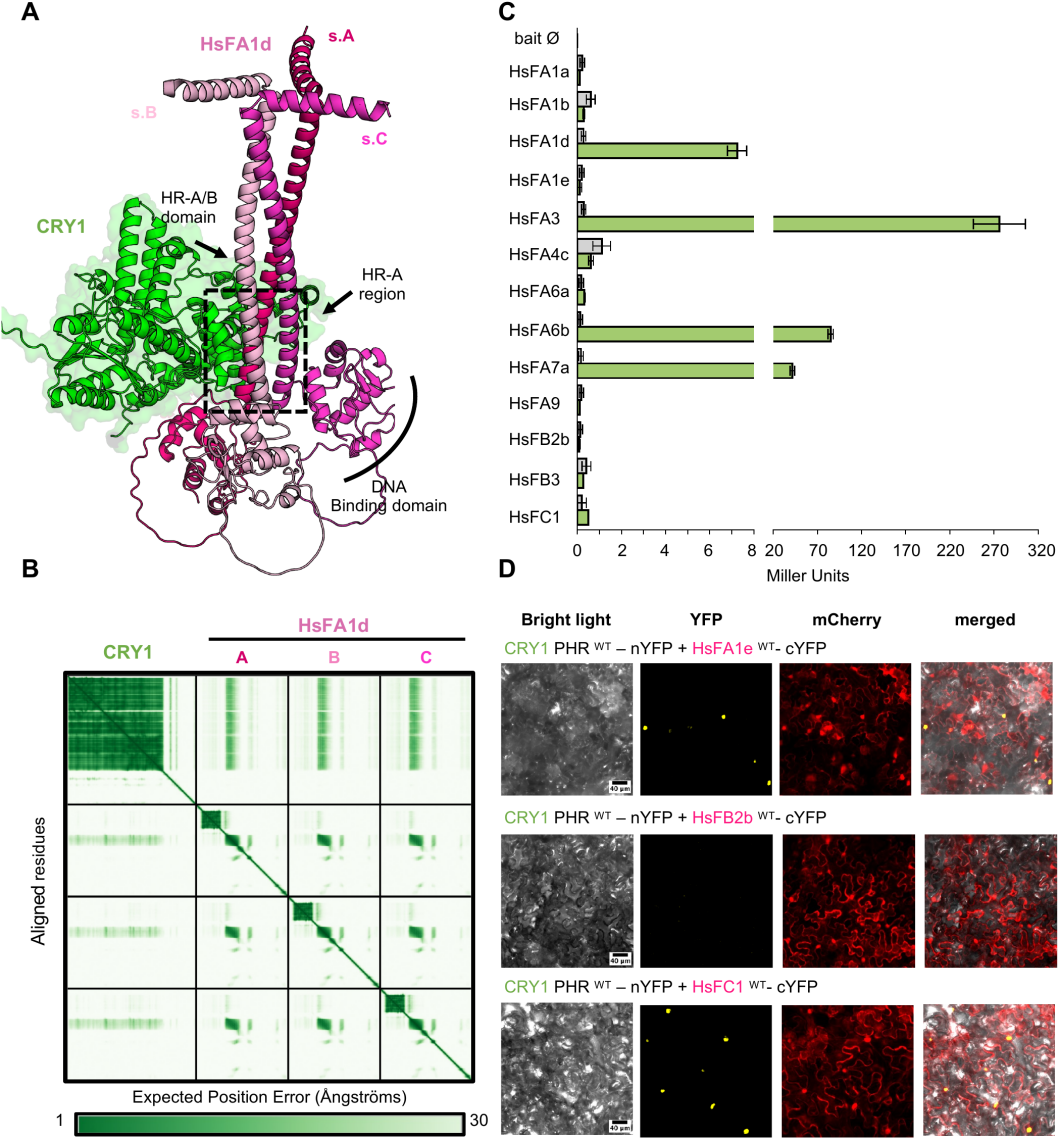
CRY1 interacts with multiple HsFs. **(A)** Structural interaction of CRY1-HsFA1d predicted by AlphaFold v3. Monomeric CRY1 is represented in green and the HsFA1d trimer in pink with each subunit (s.) of HsFA1d is represented by a different tone (subunit A: magenta; subunit B: purple; and subunit C: pale pink). CRY1 residues (509–681 aa) and HsFA1d residues (1–50 aa and 242–485 aa) are predicted to be disordered and omitted for clarity. **(B)** The predicted aligned error plot obtained by AlphaFold for the CRY1-HsFA1d model suggests an interaction between 3 monomers of HsFA1d (1–190 aa) and a monomeric CRY1 (1–508 aa). **(C)** Yeast 2-hybrid assay for CRY1-HsF interactions. Whereas 21 sequences were used *in silico* for monomeric structure prediction or interaction modeling with CRY1, only 13 of them, covering all classes, were tested *in vitro* in yeast two-hybrid assays. Precisely, interaction was assessed between pGADT7 (AD), CRY1^WT^, and pGBKT7 (BD), HsFA1a, HsFA1b, HsFA1d, HsFA1e, HsFA3, HsFA4C, HsFA6a, HsFA6b, HsFA7, HsFA9, HsFB2b, HsFB3, HsFC1. Bars show mean ± Standard deviation (n=3). For each HsF bait combination, empty prey negative controls are colored in grey. **(D)** Bimolecular fluorescence complementation assay between CRY1 and a selected subset of HsFs in *N. benthamiana* mesophyll. This assay shows interaction between CRY1^PHR WT^-nYFP and HsFA1e^WT^-cYFP, HsFB2b^WT^-cYFP or HsFC1^WT^-cYFP. Controls for bimolecular fluorescence complementation assay are presented in **Supplementary Figure S11**.

Given the previously observed interaction between CRY1 and HsFA1d, as well as the diverse roles of HsF proteins in mediating thermotolerance (Gao et al., 2023), we investigated whether all members of the *Arabidopsis thaliana* HsF family interact with CRY1 focusing first on identifying the structural differences and similarities between HsFs proteins. Comparative analysis of the structures predicted using AlphaFold of all 21 HsF proteins revealed two conserved α-helical domains present across the family: the N-terminal DNA-binding domain (DBD) and the HR-A/B oligomerization domain (**Supplementary Figure S1**). The Predicted Alignment Error plot (PAE) and the Predicted Local Distance Difference Test (pLDDT) values indicate a high level of confidence in the monomeric models around these domains (**Supplementary Figures S2**, **S3**). Sequence conservation among Arabidopsis HsF family members further highlights the high conservation in the DBD and partial conservation in the HR-A/B domain, a region that mediates trimerization (**Supplementary Figure S4**). Class A differ markedly from classes B and C by their physiological functions of transcriptional activation. Class A members notably contain activations motifs in their C-terminal region (**Supplementary Figure S4**) which are lacking in class B and class C proteins (Nover et al., 2001). AlphaFold predictions for individual HsF proteins revealed significant structural divergence among HsFs proteins. Class B members differ from class A and C HsF proteins in protein length, the presence of a long intrinsically disordered region linker between the DBD and the HR-A/B domain (**Supplementary Figures S1**–**S3**), and variable insertions/deletions around the HR-A/B domain (**Supplementary Figure S4**). PAE and pLDDT values from AlphaFold predictions for HsFs homotrimers highlight the boundaries of the HR-A/B alpha helix involved in HsFs trimerization (**Supplementary Figures S5**, **S6**). As mentioned before, this trimerization is required for the activity of HsF proteins (Ohama et al., 2016), and for their interaction with CRY1 (Gao et al., 2023). *In silico* prediction of CRY1–HsF complexes showed similar, stable interactions notably characterized by low PAE values between residues of CRY1 and HsF, only for the HsFA1s subfamily members composed of HsFA1a, HsFA1b, HsFA1d, and HsFA1e (**Figures 1A, B**; **Supplementary Figure S7**). All other 17 HsF proteins failed to produce stable interaction predictions (**Supplementary Figure S8**). Structural models of the CRY1–HsFA1s complexes reveals a rigid CRY1 PHR domain that engages in specific contacts with the structured HR-A region of the HR-A/B domain composed of residues 160-190 (**Figure 1A**; **Supplementary Figure S7**), while the C-terminal tail of CRY1 remains disordered. Importantly, while the PHR domain alone is sufficient to interact with HsFA1d in Y2H assays (**Supplementary Figures S9A, B**), this interaction is abolished in the absence of the HR-A/B helix (160–220 aa), as deletion of this domain results in a complete loss of binding in BiFC assays (**Supplementary Figure S9B**), consistent with the important role of the HR-A/B region to stabilize a functional CRY1–HsFA1d complex (Gao et al., 2023). Based on these findings, we performed subsequent experiments using a truncated CRY1 PHR domain (residues 1–508). Y2H assays were conducted with a subset of HsF proteins spanning all three classes. Strikingly, interaction with CRY1 was detected with six different HsFs: HsFA1d (7 Miller units (MU)), HsFA3 (250 MU), HsFA6a (<0.5 MU), HsFA6b (45 MU), HsFA7a (25 MU), and HsFC1 (<0.5 MU). In contrast, no interaction was observed with HsFA1a, HsFA1b, HsFA1e, HsFA4c, HsFA9, HsFB2b, and HsFB3 (**Figure 1C**). Multiple sequence alignment of the 21 HsF proteins revealed the conservation of the HR-A sequence among the family, with short insertions or deletions along the HR-A/B domain. In class B, some of the insertions are positioned adjacent to the CRY1-binding interface and may sterically hinder or disrupt interaction with the CRY1 PHR domain (**Supplementary Figure S4**). To further validate these findings, BiFC assays were performed with representative HsF proteins from classes A (HsFA1e), B (HsFB2b) for which no interaction was detected in Y2H and C (HsFC1), the only member of class. These results suggest that HsFA1e and HsFC1 form stable interactions with CRY1 in the nucleus, while HsFB2b exhibits no detectable interaction (**Figure 1D**). This may also suggest that activator HsFs of class A are preferred partners for interacting with CRY1, along with HsFC1. This last protein is considered as an activator like members of class A, however it lacks the activation motifs and thus cannot initiates transcription on its own (Jacob et al., 2017). In contrast, repressive Class B members do not appear to interact with CRY1.

### 3.2 Structural analysis of the predicted CRY1-HsFA1d interaction

**Figure 2:**
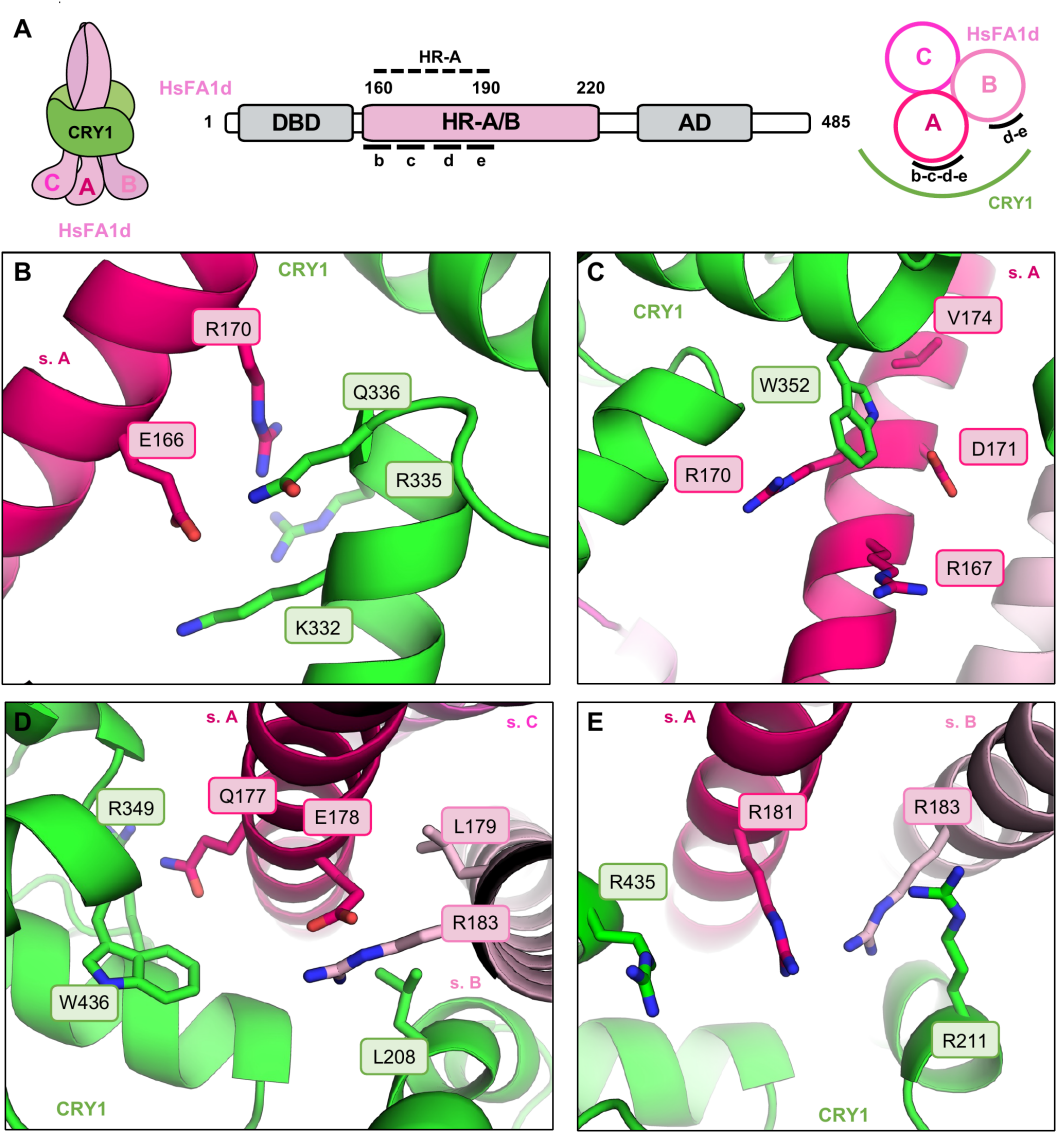
Four contact zones between HsFA1d and CRY1 are predicted by AlphaFold. **(A)** Left: Schematic representation of the CRY1-HsFA1d interacting model. Middle: Primary structure of Arabidopsis HsFA1d where domains are illustrated by boxes. Right: Transversal view of the schematic model. Contacts with CRY1 are localized along the HR-A region of HR-A/B domain and indicated by the corresponding letter of the following structure figures. CRY1 is shown in light green, and each subunit (sb) of the HsFA1d trimer is labeled with a distinct letter. **(B-D)** Close-up views highlighting the interaction regions with select contacts: **(B)** The N-terminal region of the helix. **(C)** The central region of the helix. **(D)** The C-terminal region. **(E)** the extremity of the C-terminal region of HsFA1d helix.

HsFA1d constitutes the only members of the HsF proteins family for which interaction with CRY1 was consistently detected in both Y2H and BiFC assays (**Figure 1B**; **Supplementary Figure S9**) and supported by *in silico* structural modeling (**Figure 1A**). We therefore utilized the AlphaFold-predicted structure of the CRY1–HsFA1d complex as a structural framework to identify and characterize the molecular determinants of the interaction. The model reveals that the CRY1 PHR domain binds extensively to the HR-A helical region of HsFA1d, which mediates trimerization. Specifically, CRY1 engages with the entire length of the HR-A region of subunit A, while contacting only the C-terminal HR-A of subunit B (**Figure 2A**). Key interaction residues include eight conserved residues in the HsFA1d HR-A region: E166, R167, R170, D171, V174, Q177, E178, and R181, and five residues in the CRY1 PHR domain: R211, K332, R335, W352, and R435 involved in electrostatic and hydrophobic contacts. At the N-terminal face of the HsFA1d helix, the model predicts that E166 forms a salt bridge with CRY1^K332^ and participates in hydrogen bonding interactions with both K332 and Q336 of CRY1 (**Figure 2B**). On the opposite face, HsFA1d^R170^ (subunit A) appears in proximity to CRY1^R335^. This configuration may be due to a local modeling discrepancy, yet HsFA1d^E166^ also forms a salt bridge with CRY1^R335^, suggesting a finely tuned electrostatic balance that stabilizes the interaction interface. CRY1^W352^ engages in π–cation interactions with both R167 and R170 of HsFA1d (subunit A), and D171 forms a hydrogen bond with the indole ring of W352 (**Figure 2C**). Further along the helix, Q177 (subunit A) extends toward CRY1^W436^, forming a hydrogen bond, and its amide group may interact electrostatically with CRY1^R349^*via* a dipole–charge interaction (**Figure 2D**). Nearby, E178 (subunit A) forms a salt bridge with R183 (subunit B), potentially contributing to the stabilization of the trimeric quaternary structure (**Figure 2D**). At the C-terminal end of the helical trimer, R181 (subunit A) and R183 (subunit B) project toward R435 and R211 of CRY1, forming a cluster of four arginine residues in proximity. This cluster may be stabilized by a stacking interaction between HsFA1d R181 and CRY1 R435 (**Figure 2E**). Notably, these interactions are highly conserved among HsFA1s proteins and semi-conserved across class A members (**Supplementary Figure S4**), which may explain both the functional specificity and the observed variation in binding affinity among HsF proteins.

### 3.3 Sequence and structure variation modulate CRY1 interaction

**Figure 3:**
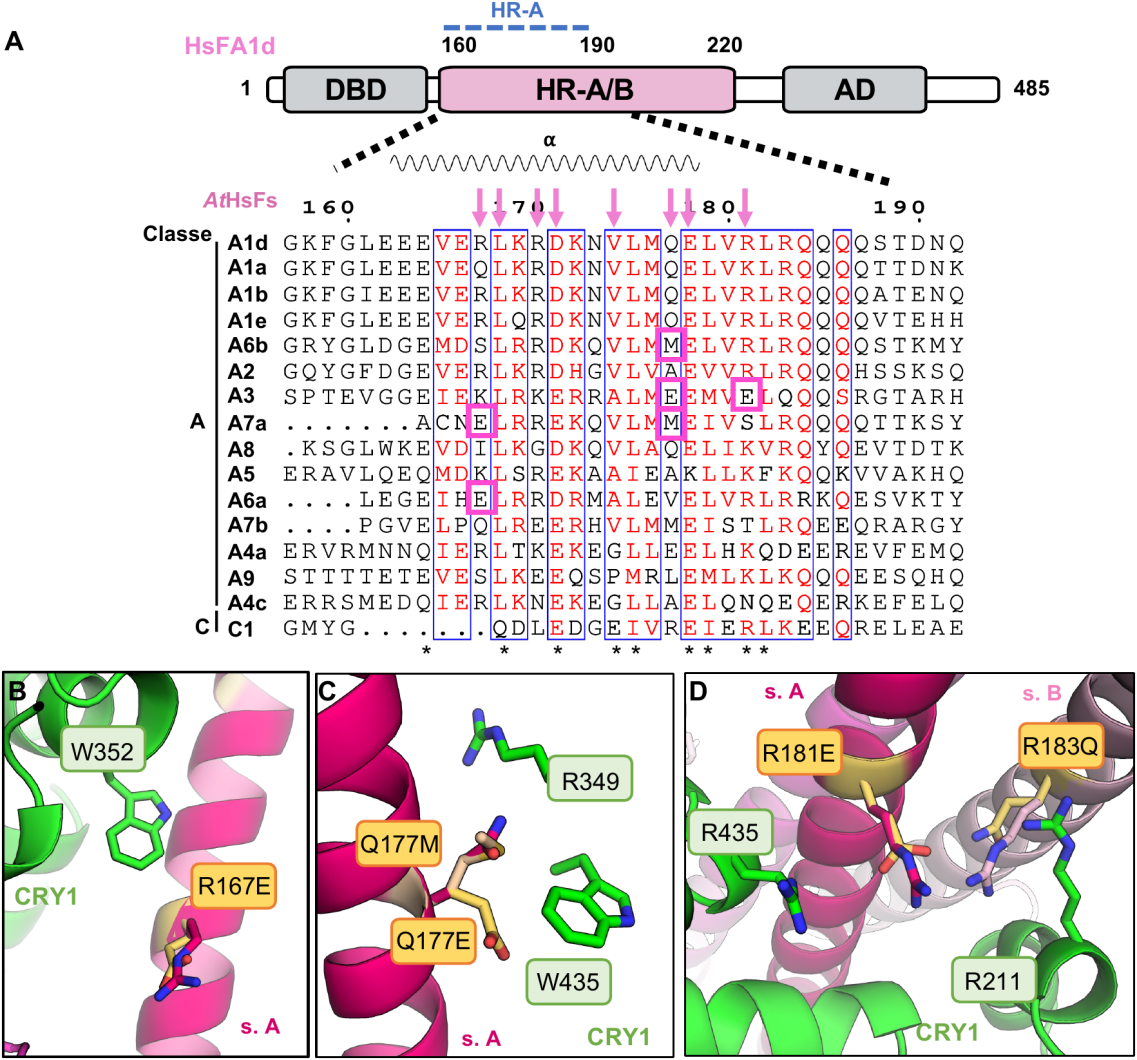
Structural plasticity in CRY1-HsFs interaction. **(A)** Sequence alignment of the HR-A/B helix for Class A and C HsFs of Arabidopsis. Top: Schematic representation of HsFA1d, where domains are illustrated by boxes. Bottom: Multiple sequence alignment for the HR-A/B helix (160–220 aa). Key HsFA1d amino acid residues predicted by AlphaFold to interact with CRY1 are indicated by pink arrows on the top of the alignment. Residues involved in protein oligomerization are indicated with an asterisk on the bottom of the alignment. **(B-D)** The model is based on the AlphaFold prediction of CRY1-HsFA1d, CRY1 residues are represented in green and HsFA1d residues from the helix in pink. **(B)** Close-up on HsFA1d R167. Arginine was manually mutated to glutamate in Pymol to mimic HsFA6a. **(C)** Close-up on HsFA1d Q177, substituted on the model by a methionine and/or a glutamate to mimic HsFA6b, HsFA7a or HsFA3. **(D)** Close-up on HsFA1d R181, arginine was manually mutated to glutamate to mimic HsFA3 E188.

BiFC and Y2H assays revealed that CRY1 interacts not only with HsFA1d but also with several other HsFAs proteins and HsFC1, indicating a broader interaction profile across the HsF family. To investigate whether sequence variation within the HR-A region modulates this interaction, we performed a sequence alignment of class A and C HsF proteins trimmed for the region corresponding to HsFA1d 160–220 aa. This sequence alignment highlights the high conservation of residues involved in both oligomerization and CRY1 PHR domain binding (**Figure 3A**). Among these, residues D171, V174, E178, and R181 of HsFA1d are consistently conserved across class A proteins and are implicated in both trimer stabilization and CRY1 binding. We hypothesized that specific amino acid substitutions in the HR-A region could modulate binding affinity to CRY1, potentially acting as gain- or loss-of-function variants in homotrimer HsF-CRY1 complexes. Among eight key residues previously identified above in the HsFA1d–CRY1 interface, we focused on those exhibiting variable conservation and thus functional potential. Towards the N-terminal part of HR-A, HsFA1d^R167^ is notably replaced by a glutamate in HsFA6a. This substitution is predicted to disrupt the interaction between R167 and CRY1 W352, which appears critical for stabilizing the interaction interface (**Figure 3B**). In the C-terminal region, Q177 is semi-conserved and replaced by a methionine in HsFA6b and HsFA7a. In HsFA3, both Q177 and R181 are substituted by glutamate residues (E184 and E188, respectively), possibly introducing negatively charged residues into an otherwise mostly electropositive region (**Figures 3A, C, D**). In the HsFA1d structural model, Q177 is positioned near R349 and W436 of CRY1. Substitution with methionine may enhance hydrophobic contacts with CRY1 W436, potentially promoting a more stable interaction through Van der Waals forces. In contrast, the substitution of Q177 with a glutamate in HsFA3 allows the formation of a salt bridge with CRY1^R349^, which may alter the electrostatic environment and modulate binding (**Figure 3C**). Most notably, in HsFA3, the natural substitution of R181 with glutamate E188 introduces a negative charge in a region typically occupied by a positively charged residue. This change enables the formation of a salt bridge between HsFA3^E188^ (subunit A) and CRY1^R435^, as well as hydrogen bonds between HsFA3^E188^ (subunit A) and HsFA3^Q190^ (subunit B), suggesting a potential for enhanced or reconfigured interactions due to the new electrostatic complementarity (**Figure 3D**). This finding suggests that specific charge reversals may generate novel interactions, potentially leading to altered binding affinity.

### 3.4 CRY1 PHR domain interact with HsFA3 HR-A region

**Figure 4:**
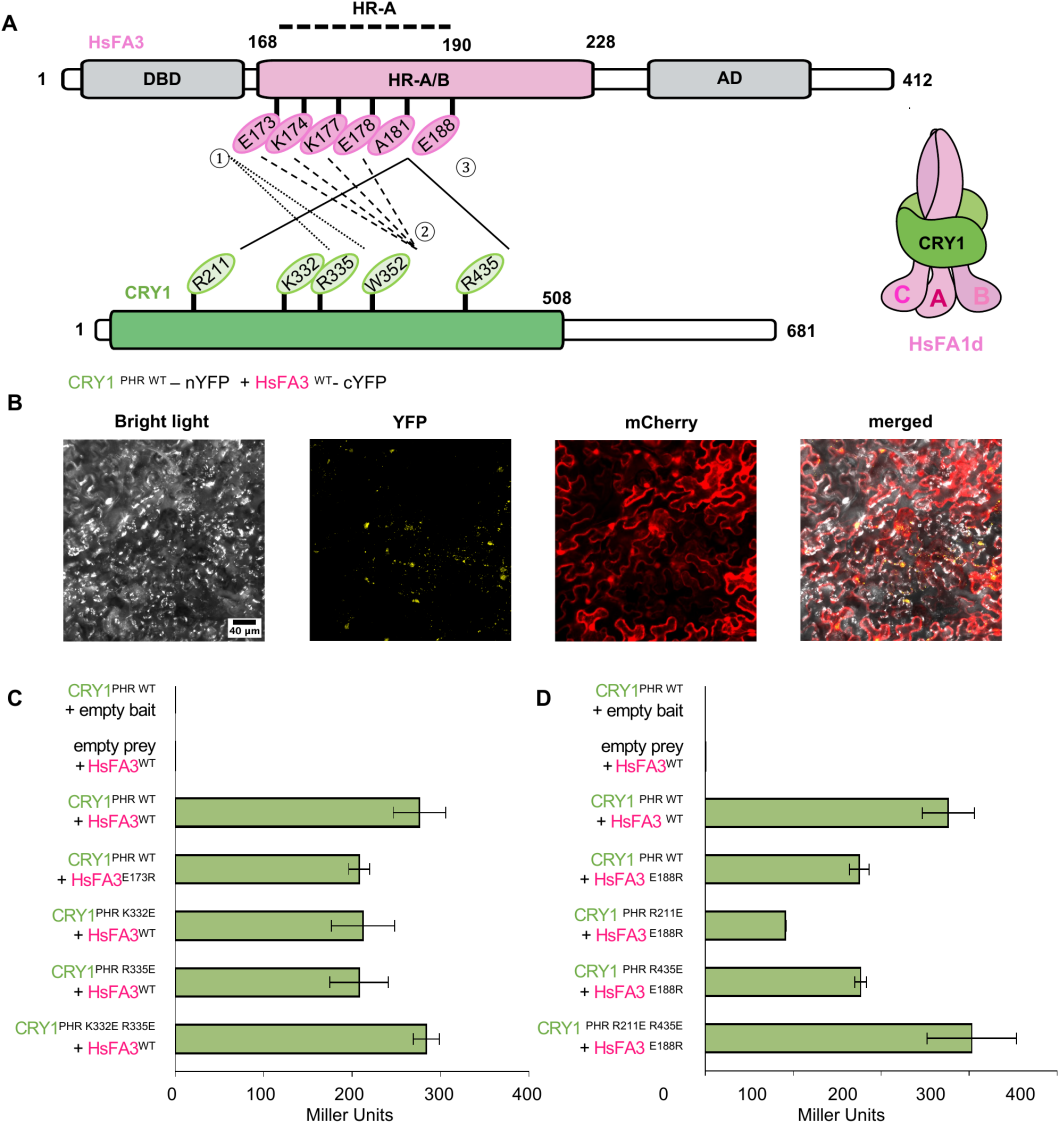
Probing of the CRY1-HsFs interface. **(A)** Schematic representation of HsFA3 and CRY1 of *Arabidospsis thaliana*. Domains are illustrated by boxes and key amino acid residues are indicated by ovals. Contacts between residues are represented in N-terminal of the helix by dotted line (1). In the middle of the helix by dashed lines (2). In C-terminal of the helix by continuous lines (3). **(B)** Bimolecular fluorescence complementation assay of CRY1-HsFA3 in *N. benthamiana* mesophyll. This assay show interaction between CRY1^PHR WT^-nYFP and HsFA3^WT^-cYFP or HsFA3^Δ(168-228)^-cYFP. **(C)** Yeast 2-hybrid CRY1-HsFA3 mutants in N-terminal of the helix. This assay shows interaction between pGADT7 (AD): empty prey, CRY1^PHR WT^, CRY1^PHR K332E^, CRY1^PHR R335E^, CRY1^PHR K332E R335E^ and pGBKT7 (BD): empty bait, HsFA3^WT^, HsFA3^E173K^ (n=3). **(D)** Yeast-two-hybrid CRY1-HsFA3 mutants in C-terminal of the helix. This assay shows interaction between pGADT7 (AD): empty prey, CRY1^PHR WT^, CRY1^PHR R211E^, CRY1^PHR R435E^, CRY1^PHR R211E R435E^ and pGBKT7 (BD), empty bait, HsFA3^WT^, HsFA3^E188K^. Bars show mean ± Standard deviation (n=3).

To validate the predicted interaction interface between CRY1 and members of the HsF family, we selected HsFA3 as a model protein, as it exhibits the highest binding activity in Y2H assays providing a strong functional basis for probing residue-level contributions. Since AlphaFold failed to predict an interaction between CRY1 and HsFA3 although HsFA3 and HsFA1d share high sequence and structure similarity on HR-A region, we generated a homology model using the Swiss-model and the CRY1–HsFA1d complex as a template (**Figure 4A**). The interaction between CRY1 and HsFA3 is supported in part by our Y2H assay (**Figure 1C**; **Supplementary Figure S10**). BiFC assays further confirmed that the CRY1–HsFA3 interaction occurs within some distinct nuclei of infiltrated mesophyll cells (**Supplementary Figures S10**, **S11**), but their distribution differs from that observed for the other HsFs tested, and resembles that seen during photobody formation (Yu et al., 2009; Yang et al., 2023). Consistent with a model where the HR-A/B region of HsFA3 interacts with CRY1, deletion of 168–228 aa of HsFA3^Δ(168–228)^ abolished interaction with CRY1 (**Figure 4B**). To further validate the homology model, we investigated the contribution of specific residues identified in the previous section to the CRY1-HsFA3 interaction. First, we focused our analysis on the N- and C-terminal ends of the HR-A region, because each extremity features at least one salt bridge, allowing charge swaps to validate the model. Substituting HsFA3^E173^ with arginine significantly reduced the interaction to CRY1 compared to the wild type (WT). Swapping the charges K332 or R335 of CRY1 to glutamate alone did not restore WT interaction levels. However, a triple charge-swap restored the interaction to WT levels (**Figure 4C**). Similarly, in the C-terminal end of the helix, substituting HsFA3 E188 with arginine reduced the interaction by approximately one-third. Individual charge reversals of CRY1 R211 or R435 to glutamate were insufficient to restore the WT level yet combining these modifications with CRY1^E188R^ restored activity to WT levels (**Figure 4D**). Computational analysis using mCSM (Pires et al., 2014) predicted minimal changes in stability for the point mutants, with ΔΔG values indicating little difference compared to the wild-type protein (see **Supplementary Table 5**).

### 3.5 Compensatory interactions at the CRY1-HsF interface

**Figure 5:**
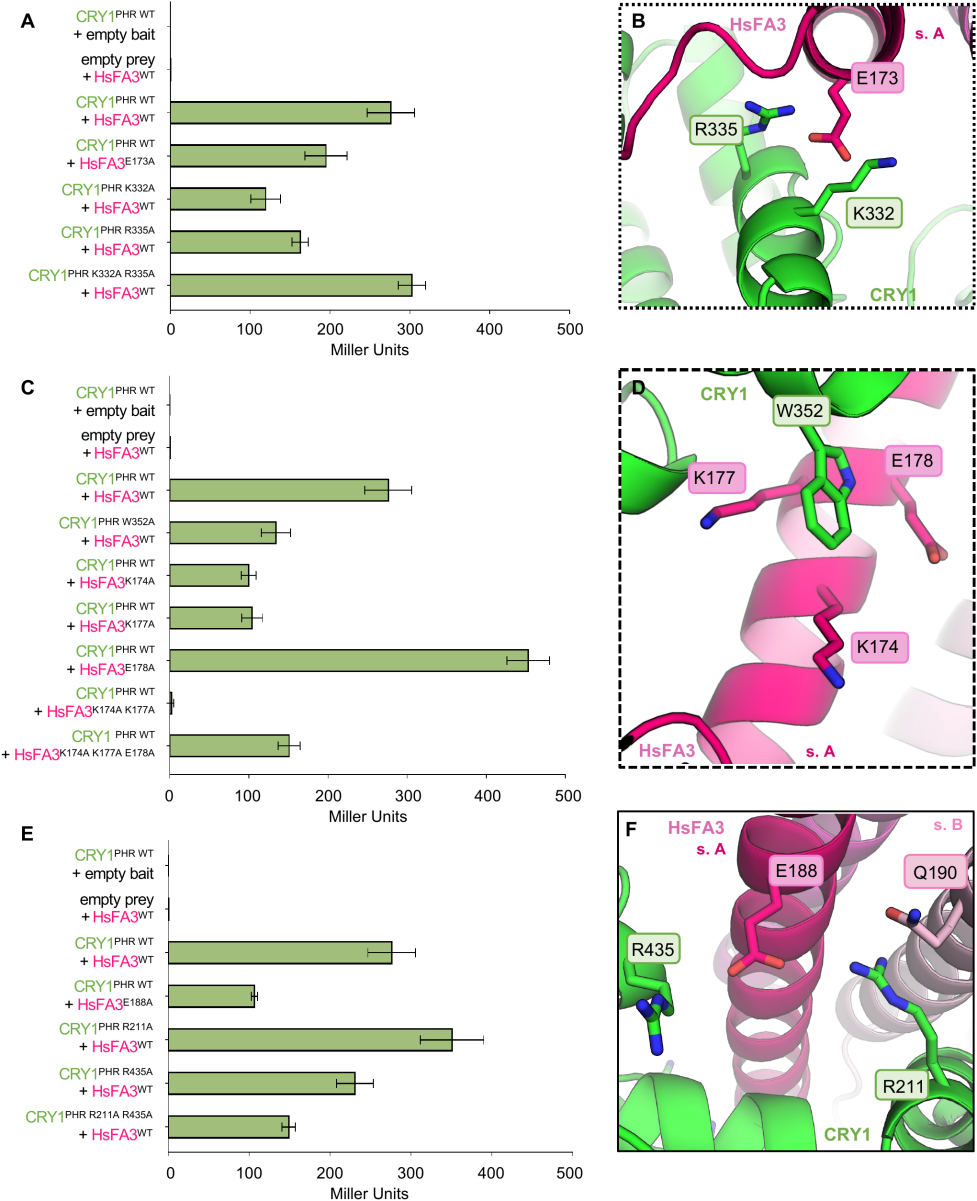
Contribution of candidate residues identified by the CRY1-HsFA3 homology model inspired by AlphaFold prediction. **(A, C, E)** Yeast-two-hybrid probing of CRY1-HsFA3 mutants. Bars show mean ± Standard deviation (n=3) and **(B, D, F)** the corresponding structural representation. **(A)** This assay shows interaction between pGADT7 (AD): empty prey, CRY1^PHR WT^, CRY1^PHR K332A^, CRY1^PHR R335A^, CRY1^PHR K332A R335A^ and pGBKT7 (BD): empty bait, HsFA3^WT^, HsFA3^E173A^**(B)** Detailed view of the HsFA3 N-terminal helix where HsFA3^E173^ is stabilized through interactions with CRY1^K332^ and CRY1^R335^. **(C)** This assay shows interaction between pGADT7 (AD): empty prey, CRY1^PHR WT^, CRY1^PHR W352A^ and pGBKT7 (BD): empty bait, HsFA3^WT^, HsFA3^K174A^, HsFA3^K177A^, HsFA3^E178A^, HsFA3^K174A K177A^, HsFA3^K174A K177A E178A^. **(D)** Structural interaction between CRY1^W352^ and HsFA3^K174^, HsFA3^K177^ and HsFA3^E178^. **(E)** This assay shows interaction between pGADT7 (AD): empty prey, CRY1^PHR WT^, CRY1^PHR R211A^, CRY1^PHR R435A^ and pGBKT7 (BD): empty bait, HsFA3^WT^, HsFA3^E188A^. **(F)** Detailed view of the HsFA3 C-terminal helix where residues HsFA3^E188^ is stabilized through interactions with CRY1^R211^ and CRY1^R435^.

The CRY1–HsFA3 homology model generated using CRY1-HsFA1d as a template, combined with the HsFs sequence alignment, enabled the identification of several candidate residues in HsFA3 for alanine substitution (**Figure 3A**, **4C**): E173, K174, K177, E178, A181, E184, E185, and E188. All these residues are located within the HR-A region of HsFA3. In the N-terminal region of the HsFA3 helix, a E173A substitution leads to a 29% decrease of the interaction with CRY1 compared to the WT (**Figures 5A, B**). Analysis of the model suggests that substituting CRY1^K332^ to an alanine could disrupts both the salt bridge and hydrogen bonds with HsFA3^E173^, and it experimentally results in a 56% reduction relative to WT. This difference may be partially offset by HsFA3^R177^, which could shift to a more favorable position to form a new hydrogen bond with the amide group of Q336. R335A may disrupt a salt bridge with HsFA3^R177^, and this effectively results in Y2H assays in a 41% decrease in Miller units (**Figures 5A, B**). This highlights a synergistic contribution of CRY1 K332 and R335, stabilizing the local conformation of E173, thereby enhancing overall interface stability. In the central region of the helix, W352A reduces the interaction by 51% compared to WT. Based on the model, this substitution could disturb π–cation interactions with HsFA3 K174 and K177. Individual substitution of each lysine to alanine residues yields to similar values to CRY1^W352A^, with decreases of 64% and 62% observed by Y2H, respectively. Interestingly, HsFA3^E178A^ enhances the interaction, doubling Miller unit levels relative to the WT. This mutation is predicted to disrupt a π–anion interaction with CRY1^W352^, which may provide conformational rigidity. The conformational freedom resulting from the E178A mutation may facilitate W352 movements. The importance of both lysine residues is supported by the fact that the complete inactivity of the HsFA3^K174A K177A^ double mutant in the Y2H assay (values under the detection limit). HsFA3^K177^ is also located near CRY1 R335 and Y383. However, a triple substitution HsFA3^K174A K177A E178A^ partially restores the interaction, potentially through the formation of new hydrophobic contacts with W352 (**Figures 5C, D**). In the C-terminal region of the HsFA3 helix, the E188A mutation leads to a 61% decrease in interaction relative to the WT. The CRY1^R211A^ substitution increases Miller units by 27%, possibly due to a conformation that stabilizes the salt bridge formed between CRY1^R435^ and HsFA3^E188^. Hydrophobic interaction can also be formed by CRY1^R211A^ with the nearby HsFA3^Q190^ of subunit B. HsFA3^E188^ of subunit A and HsFA3^Q190^ of subunit B are predicted to form hydrogen bonds that stabilizes the trimer by linking monomers together. The CRY1^R435A^ substitution causes a 12% reduction of the reporter gene expression compared to WT, which could be due to disruption of the salt bridge between HsFA3^E188^ and CRY1^R435^, although the surrounding environment remains otherwise unchanged (**Figures 5E, F**). Overall, these results suggest that three regions of interaction contribute to efficient CRY1-HsFA3 interaction. Alanine mutations thus revealed select cooperations between residues as well as compensatory interactions. With the notable exception of CRY1 R335A which stands close to the strongly destabilizing threshold, all mutations tested in this section are predicted to be either nondestabilizing or moderately destabilizing according to the stability change value ΔΔG (Pires et al., 2014) reported in the **Supplementary Table 5**.

### 3.6 Development of HsFs mutants with increased interaction to CRY1

**Figure 6:**
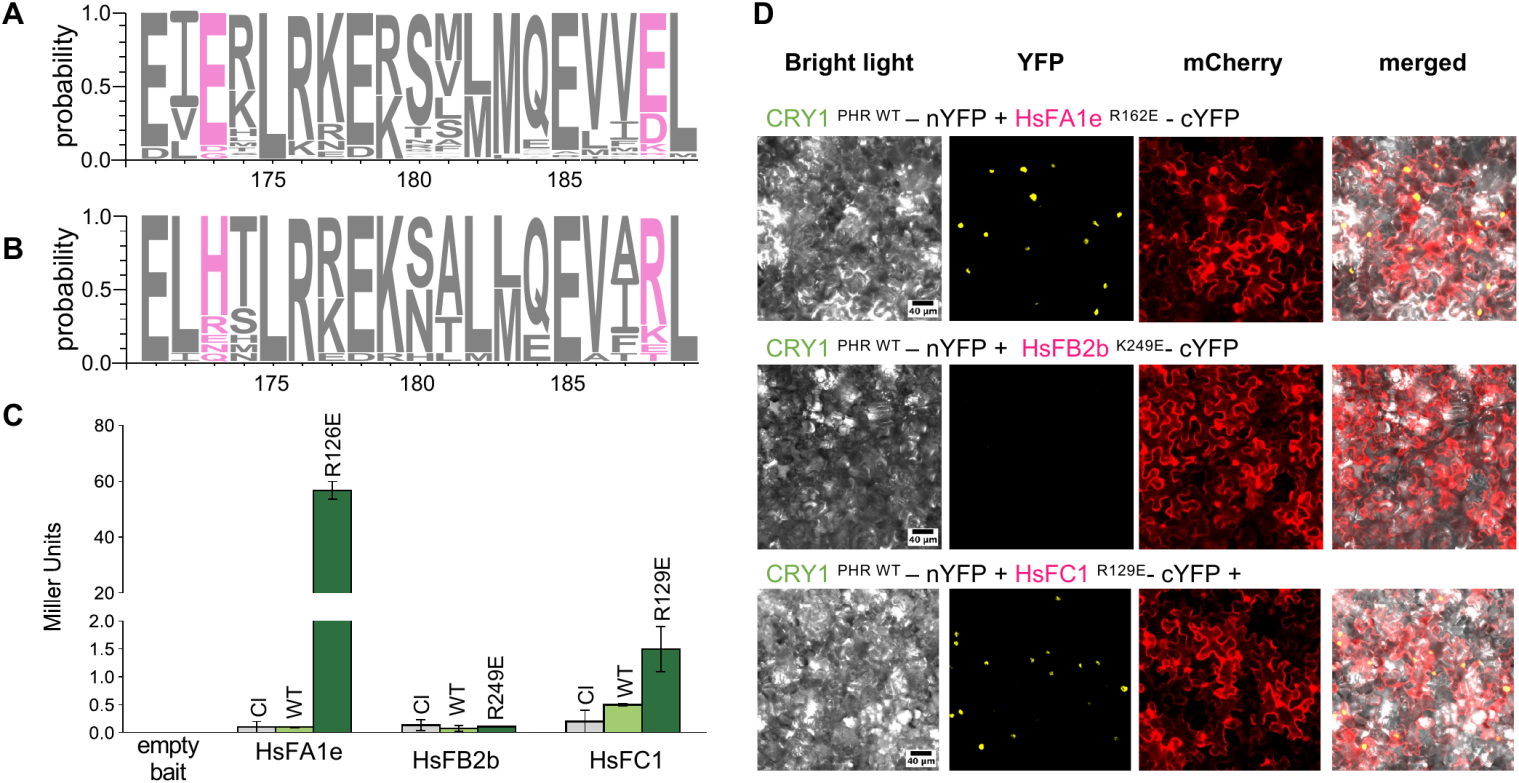
New HsFs gain of function mutants inspired by HsFA3 natural variability. **(A)** Sequence logo of monocotyledon’s HsFA3. **(B)** Sequence logo of dicotyledon’s HsFA3. Sequences are centered on the HR-A region of AtHsFA3. Letter size indicates probability of amino acid at a given position. Amino acids corresponding to position 173 and 188 for Arabidopsis HsFA3 are indicated in pink. **(C)** Yeast-two-hybrid CRY1-HsFs gain of function. This assay shows interaction between pGADT7 (AD): empty prey, CRY1^PHR WT^ and pGBKT7 (BD): empty bait, HsFA1e^WT^, HsFA1e^R126E^, HsFB2b^WT^, HsFB2b^K249E^, HsFC1^WT^, HsFC1^R129E^. Bars show mean ± Standard deviation (n=3). For each HsF bait combination, empty prey negative controls are colored in grey. Wild-type is colored in light green while mutations are colored in dark green. **(D)** Bimolecular fluorescence complementation assay of CRY1-HsFs in *N. benthamiana* mesophyll. This assay shows interaction between CRY1^PHR WT^-nYFP and HsFA1^eR126E^ -cYFP, HsFB2b^K249E^-cYFP HsFC1^R129E^ -cYFP.

To validate the predicted interaction interface of the CRY1-HsFA3 complex, we performed charge-swap mutagenesis at key salt-bridge residues in the N- and C-terminal domains, focusing on HsFA3 E173 and E188 residues predicted to stabilize the interaction interface based on AlphaFold modeling and sequence conservation (**Figure 4**). Mutation of either E173 or E188 to a positively charged residue significantly impaired binding affinity in Y2H assays, confirming the functional importance of these negatively charged residues in stabilizing the complex (**Figure 4**). Sequence alignment analysis across HsFs-type proteins revealed semi-conservation of E173 and E188 in *Arabidopsis thaliana* HsFA3, with a high degree of conservation in dicotyledons, with 90% and 70% prevalence, respectively (**Figure 6A**). Notably, these positions are frequently substituted with positively charged residues (histidine, lysine, or arginine) in monocotyledonous species, suggesting a potential evolutionary shift of HsFA3 in interaction mechanisms (80% prevalence, **Figure 6B**). Based on these findings, the contribution of a negative charge at this position was evaluated in terms of its impact on the CRY1-HsFs interaction specificity. We tested whether introducing charge swap mutation to certain HsF proteins could increase their interaction with CRY1. Precisely, HsFA3 mimicking mutations where used to assess interaction competence in candidates from classes A, B, and C proteins previously found to barely interact - or not - with CRY1 in Y2H assays. Y2H assays revealed a strong gain-of-function effect in HsFA1e^R126E^ and HsFC1^R129E^, with interaction levels increasing by approximately 40-fold and 10-fold, respectively, compared to the WT control (**Figure 6C**). In contrast, no significant reporter activity was observed in HsFB2b^K249E^, suggesting that the mutation at this site does not rescue interaction and may reflect a class-specific structural or regulatory constraint. Alternatively, the enhanced interaction could remain undetectable within the experimental sensitivity. These results were corroborated by BiFC, which confirmed the formation of HsF–CRY1 complexes in HsFA1e^R126E^ and HsFC1^R129E^, as evidenced by robust fluorescence signal *in planta* (**Figure 6D**). Overall, these results indicate that introducing a negative charge at HsFA3 position 188 in other HsF members triggers an increase in the interaction with CRY1. Importantly, mCSM calculations suggest that these point mutations are unlikely to significantly affect protein stability (**Supplementary Table 5**).

## 4 Discussion

Our results suggest that CRY1 is engaged in a broad network of interactions across the HR-A region of HsF proteins, revealing a previously underappreciated complexity in the regulation of light-dependent heat responses. Beyond the previously identified interaction between CRY1 and HsFA1s subfamily (Gao et al., 2023), we observed that CRY1 interacts with multiple other HsFs proteins across both Class A and C. As described by (Gao et al., 2023), the CRY1 PHR domain (1–490 aa) interacts with HsFA1d (1–220 aa). They highlight a possible contribution from the disordered C-terminal tail of CRY1. Our data expand on these findings by revealing a unique interface between the PHR domain of CRY1 and the HR-A region of the HR-A/B domain of HsF proteins. The semi-conservation of residues along the HR-A region appears to modulate interaction with CRY1. In particular, a subtle amino acid changes within the HR-A domain appears to strongly influence the strength and nature of the CRY1 interaction, providing a plausible molecular basis for HsF proteins-specific functional diversification. HR-A and HR-B confer an oligomeric structure that properly positions the DBD domains to bind to HSEs. The flexibility of the linker between the DBD and the HR-A/B domain enables adaptation to variable HSE geometries (Feng et al., 2021), and plays a critical role in heat response activation, as well as in transcriptional positioning and efficiency (Sorger and Nelson, 1989). Interaction with CRY1 enhances HsFA1d’s affinity for HSEs and increases the number of genes targeted by the HsFA1d-CRY1 complex (Gao et al., 2023). Thus, CRY1 may contribute to DBD positioning by modulating the organization of disordered linker, enabling contact with new HSEs in response to stress. The insertion upstream of HR-A in class B HsFs (**Supplementary Figure S4**) could disrupt this mechanism. Our data indicate that HsFA3 exhibits stronger interaction affinity compared to members of the class A subfamily (**Figure 1B**). Based on these properties, HsFA3 was selected as a model system for probing residue-level contributions. Our results reveal that HsFs are stabilized by two key salt bridges formed by HsFA3^E173^ and HsFA3^E188^ interacting respectively with positive patches composed of (K332, R335) and (R211, R433), creating an electrostatic complementarity that enhances overall interface stability with CRY1. At the heart of this interaction, tryptophan CRY1^W352^ participates in charged interactions with K174, K177, and E178 of HsFA3, underscoring the importance of interactions among this interacting surface. Structural studies reveal that CRY1 forms oligomers that implicate a through a conserved π-cation interaction between CRY1^W352^ and CRY1^R442^ (Shao et al., 2020). Dimerization may thus results in the masking of the HsF binding interface by a CRY1 protein whereas photoactivation, by triggering monomerization, may facilitate interaction with proteins partner (Shao et al., 2020). CRY1-HsFA3 interactions are strongly dependent on the conservation of AtHsFA3 residues at position corresponding to E173, K174, K177 and E188 which are preserved in dicotyledonous HsFA3s, whereas they are frequently substituted in monocotyledonous. This evolutionary divergence suggests functional adaptation: in particular, the negative charge at the C-terminus of HsFA3 present primarily in dicots may play a key role in stabilizing the interface. In Arabidopsis, HsFA3 stands out as the only member of the HsF family to possess a negative charge in its C-terminal region, a remarkable evolutionary feature that may play a key role in stabilizing the interaction with CRY1 and in fine-tuning the stress response. This structural peculiarity fits into a broader context of functional diversification observed in dicots, which exhibit a greater variety of HsF proteins and have evolved for specialized roles in various physiological processes (Xu et al., 2020). The *in vivo* CRY1-HsFA3 interaction, demonstrated by BiFC, suggest that CRY1-HsFA3 interaction mainly occurs in the nucleus as puncta, a signal distribution not observed as homogenic as seen in other HsFs such as AtHsFA1s (Gao et al., 2023) and resembles features previously associated with nuclear bodies. This specificity suggests that the CRY1–HsFA3 pathway may be specialized in coordinating nuclear regulatory responses. Previous studies (Yu et al., 2009; Yang et al., 2023) have shown that similar structures correspond to photo bodies formed upon CRY1 accumulation, either awaiting degradation by the proteasome or functioning as transient stress structures that positively influence gene expression. AtHsFA3 forms heterotrimers with seven other HsFAs proteins, leading to enhanced transcription and promoting heat response sensitivity and specificity (Friedrich et al., 2021). Our analysis was restricted to HsFs homotrimers, where some possible new interactions by arginine stacking from R181 (subunit A) and R183 (subunit B) observed on HsFA1d homotrimers predicted structure (**Figure 2E** and **3D**) could be replaced by strong electrostatic interaction formed by HsFA3^E188^ (subunit A) and HsFA1d^R183^ (subunit B), reenforcing HsFs trimerization and CRY1 interaction in HsFA1d-HsFA3 hetero-trimer context. Furthermore, position 188 represents a prime target for developing rational gain-of-function mutants in monocotyledons such as *Oryza sativa* (**Supplementary Figures S12**, **S13**), or in other HsF members of *Arabidopsis* (e.g. HsFA1e), to regulate specific stress responses. The results from the HsFA1e^R162E^ and HsFC1^R129E^ mutants for which mutation at position 188 strongly increase interaction with CRY1 highlighted the potential of this residue. In contrast, the lack of response in the HsFB2b^K249E^ mutant may suggest a specific structural or functional barrier among members of the B subfamily. Alternatively, it may reflect distinct roles in stress response regulation, or a limitation of the reporter system used in our studies. Finally, while experimental confirmation of mutant protein stability would be valuable, mCSM predictions (Pires et al., 2014) indicate that most mutations tested have minimal or moderate destabilizing effects on protein stability, with R335A lying just beyond the threshold for strong destabilization. These results (**Supplementary Table 5**) overall suggests that those mutants are more likely to affect interaction rather than stability. Although our study hinted at the importance of specific interactions, understanding the complete functional consequences of these interactions will require future validation in plants.

## 5 Conclusion

In this study the CRY1–HsFA3 interaction relies on a precise network of electrostatic interactions, strongly conserved in dicots but less in monocots. This functional divergence may reflect evolutionary adaptations to different environments, where the thermal stress response is differentially orchestrated. The identification of key residues for the interaction between CRY1 and HsFs opens the way to the design or selection of functional variants with potential applications in plant biotechnology, particularly in developing plants with enhanced thermal stress tolerance.

## Data Availability

The raw data supporting the conclusions of this article will be made available by the authors, without undue reservation.
