# Supplementary material for: The CRY1-HsF predicted interaction interface serves as a molecular platform for bioengineering or selecting modulating mutants
Source: Front Plant Sci. 2025 Dec 17;16:1712571. doi: 10.3389/fpls.2025.1712571 (PMC12753876; doi:10.3389/fpls.2025.1712571)
Supplement: Supplementary file 1 [file DataSheet1.pdf]

Supplementary Material

The CRY1-HsF predicted interaction interface serves as a molecular platform for bioengineering or selecting modulating mutants

Souleïmen Jmii, William Bouard, Gabriel Marcotte, Julien Plamondon and Laurent Cappadocia

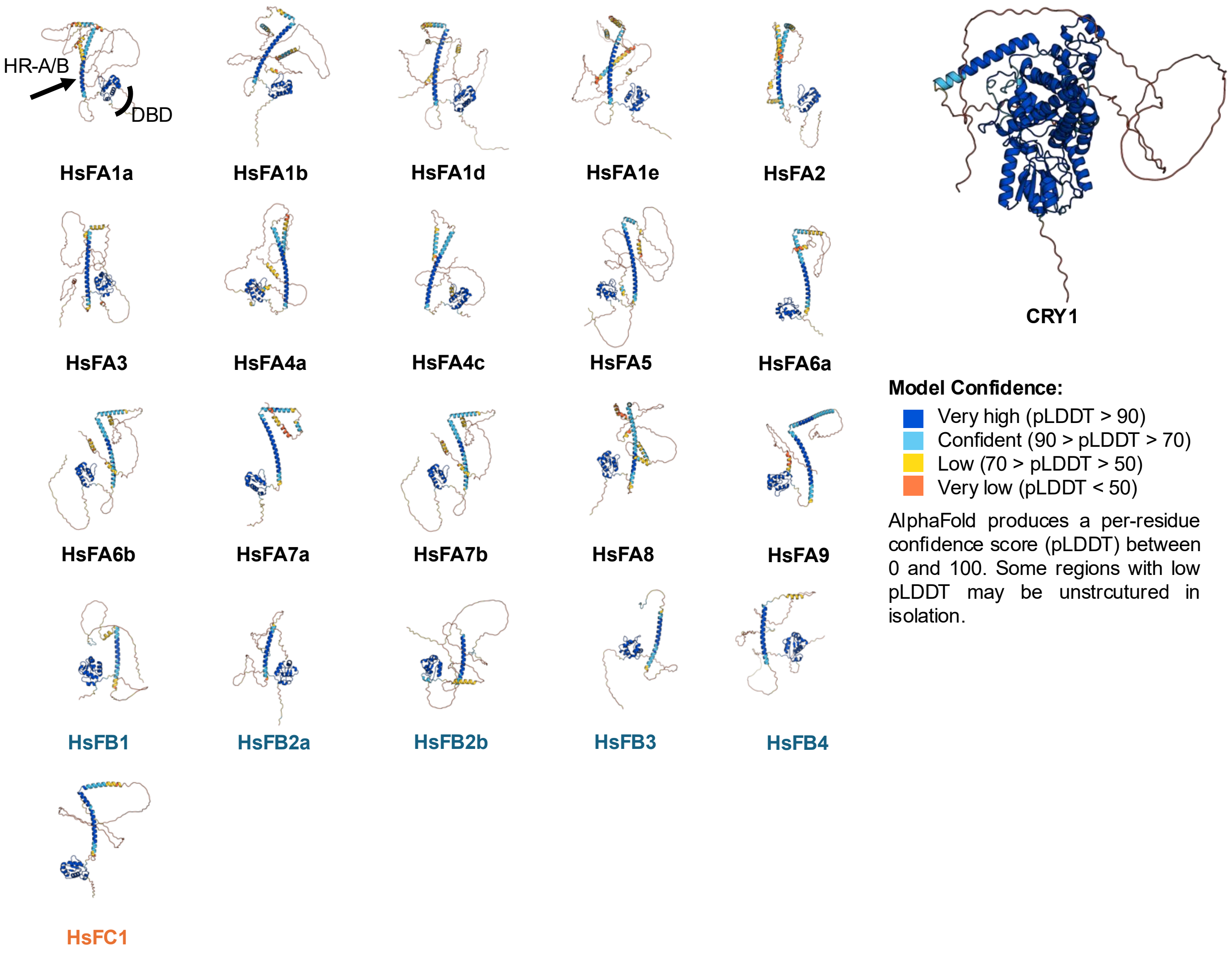

Figure S1. Predicted three-dimensional structures of CRY1 and 21 heat shock proteins from *Arabidopsis thaliana*. Proteins were modeled as monomers. The prediction reveal secondary structural elements, a rigid DNA binding domain and the alpha helix HR-A/B allowing oligomerization. The level of confidence for proteins is represented by their pLDDT values as determined by AlphaFold: very high above 90 (blue), confident between 70 and 90 (pale blue), low between 50 and 70 (yellow) and very low below 50 (orange).

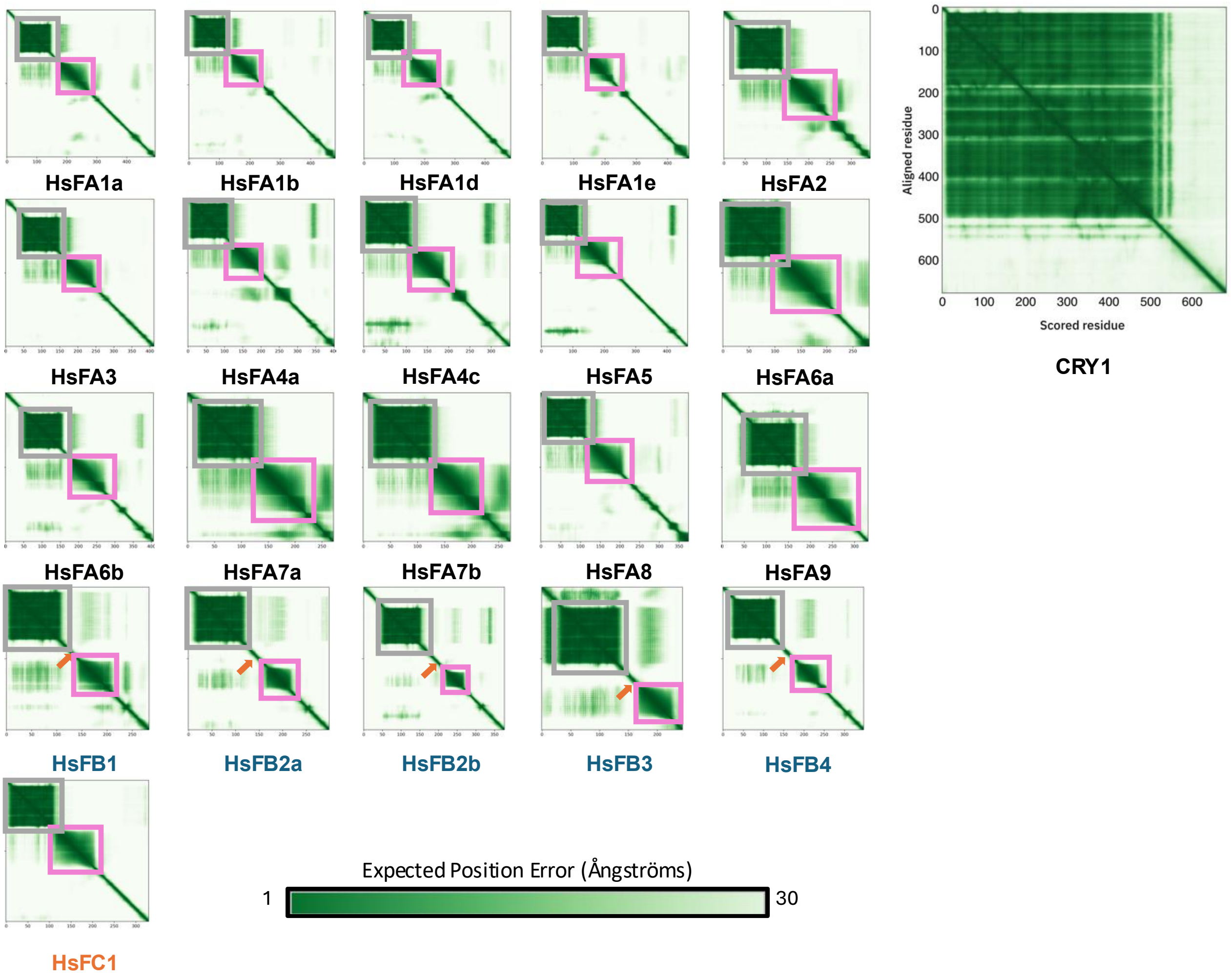

Figure S2. Predicted aligned error plot (PAE) of CRY1 and 21 monomeric heat shock proteins. The PAE diagrams were obtained using AlphaFold v3 and allow identification of protein domains and structured regions. The DNA-binding domains and the oligomerization domains are boxed in grey, and pink, respectively. An orange arrow points to the long-disordered region between the two domains in the HsF proteins of class B.

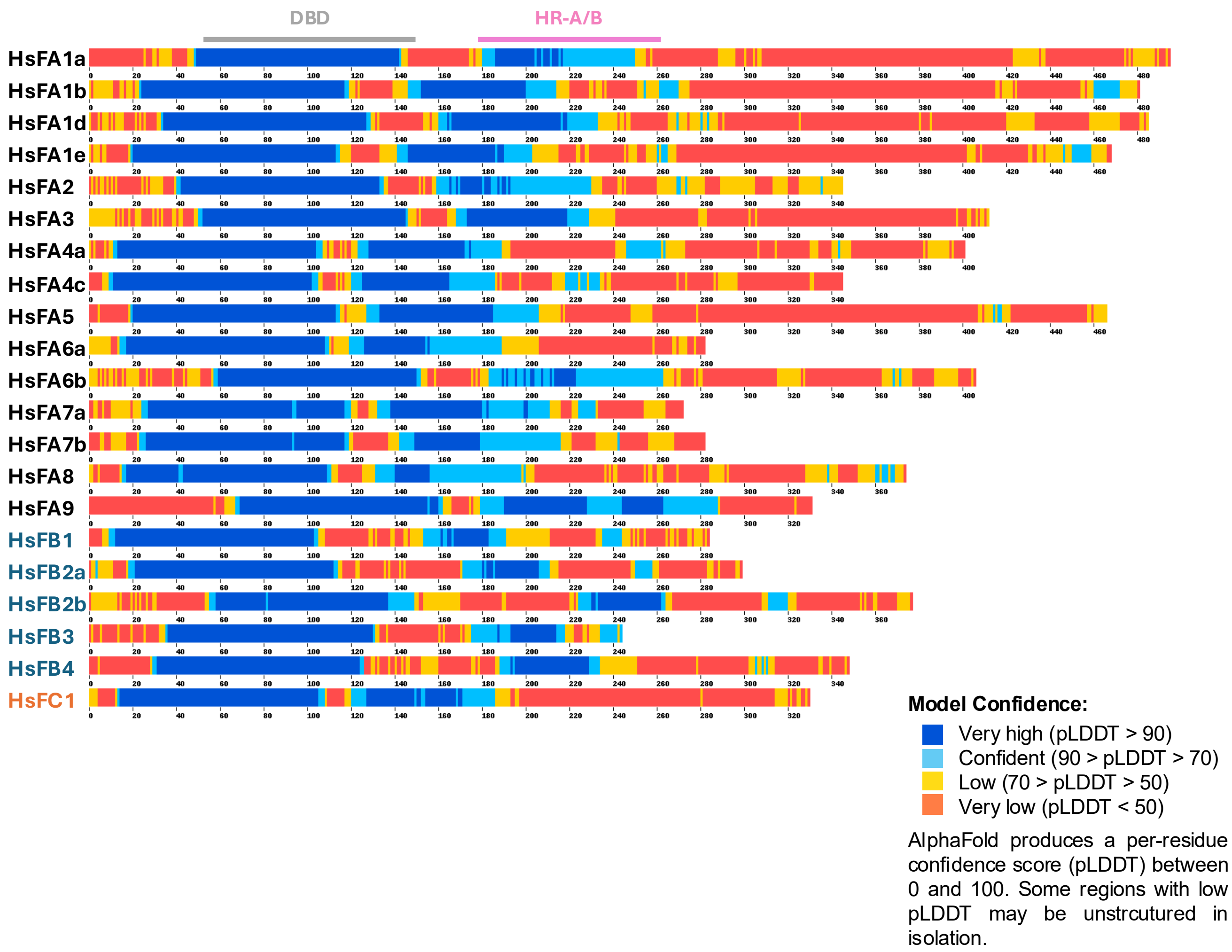

Figure S3. pLDDT values of 21 heat shock proteins from *Arabidopsis thaliana*. Proteins are represented by rectangles, colored according to their per-residue pLDDT values.

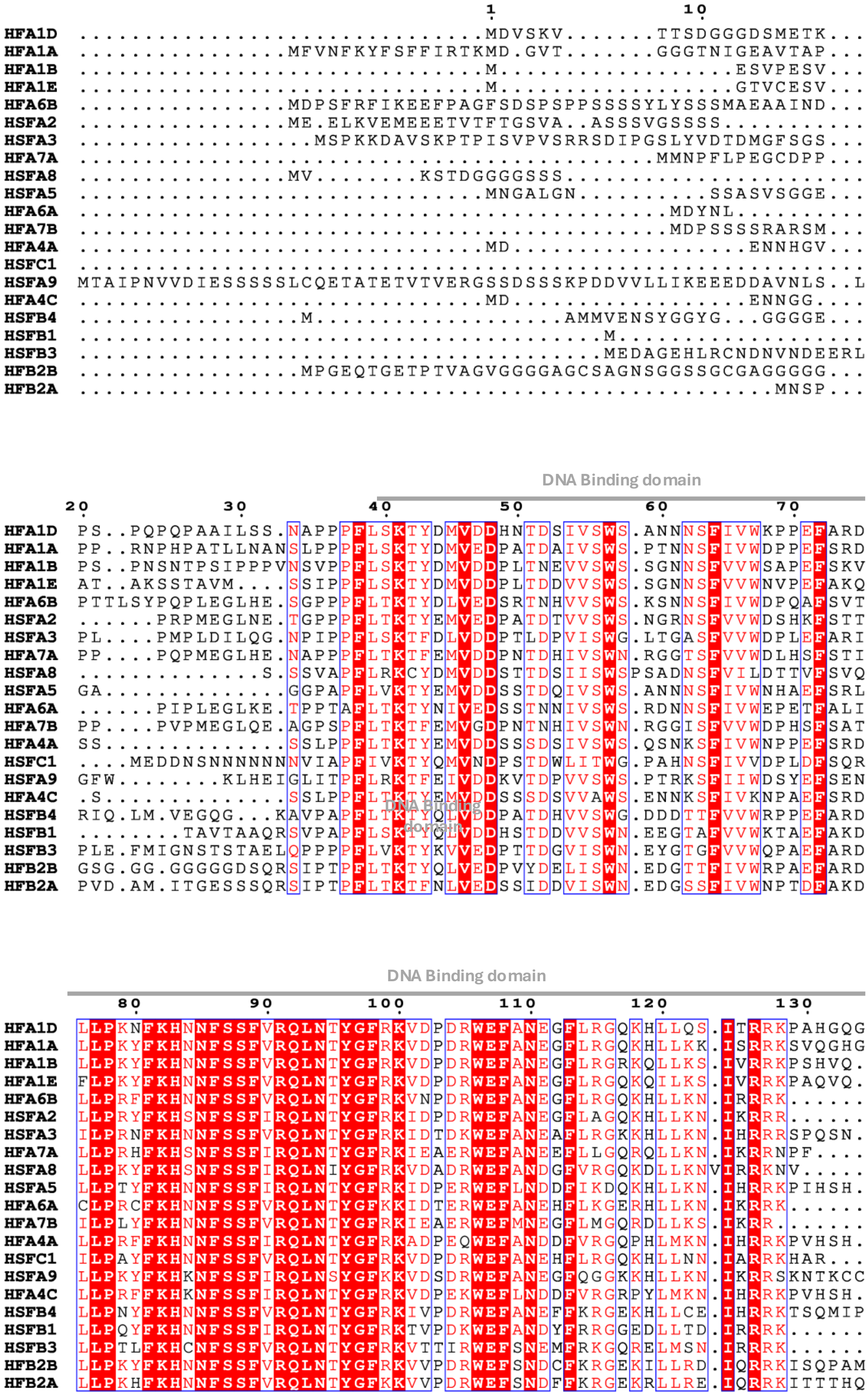
DNA Binding domain
DNA Binding domain
HFA1D
HFA1A
HFA1B
HFA1E
HFA6B
HSFA2
HSFA3
HFA7A
HSFA8
HSFA5
HFA6A
HFA7B
HFA4A
HSFC1
HSFA9
HFA4C
HSFB4
HSFB1
HSFB3
HFB2B
HFB2A

```
         140       150
HFA1D QGHQRSQ.....HSNGQNSSVSACVE..................................
HFA1A SSSSNPQSQQLSQGQGSMAALSSCVE..................................
HFA1B ...QNQQ.....QTQVQSSSVGACVE..................................
HFA1E ...P.PQ.....QPQVQHSSVGACVE..................................
HFA6B TSNNSNQMQQ.PQSSEQQSLDNFCIE..................................
HSFA2 NMGLQNVNQQ.GSGMS.......CVE..................................
HSFA3 QTCCSSTSQS...............Q..................................
HFA7A TPSSSP............SHD.......................................
HSFA8 ..QSSEQSKH.ESTSTTYAQE.......................................
HSFA5 .SHPPASS................TD..................................
HFA6A TSSQTQ............TQS.......................................
HFA7B TSSSSPPSLN.YSQSQPEAHD.......................................
HFA4A .SLPNLQAQL...........NPLTD..................................
HSFC1 ............................................................
HSFA9 NKEASTTTTE..................................................
HFA4C .SLVNLQAQ............NPLTE..................................
HSFB4 QQHSPFMSHH.HAPPQIPFSGGSFFP.LPPPRVTTPEEDH...YWCDDSPPSRPRVIPQQ
HSFB1 SVIAS.TAGK.CVVVGSPSESNSGG.........GDDHGS....SSTSSPGS.......S
HSFB3 SQHWSHNKSN.HQVVPTTTMVNQEGH.....QRIGIDHHH....EDQQSSAT.......S
HFB2B AAAAAAAAAA.VAASAVTVAAVPVVAHIVSPSNSGEE....QVISSNSSPAAAAAAIGGV
HFA2A TVVAPSSEQR.NQTM............VVSPSNSGEDNNNNQVMSS..SPSSWYCHQTKT
```

HR-A

HR-A/B domain

```
        160                 170       180       190       200
HFA1D VGKFGLE............EEVERLKRDKNVLMQELVRLRQQQQSTDNQLQTMVQRLQGM
HFA1A VGKFGLE............EEVEQLKRDKNVLMQELVKLRQQQQTTDNKLQVLVKHLQVM
HFA1B VGKFGIE............EEVERLKRDKNVLMQELVRLRQQQQATENQLQNVGQKVQVM
HFA1E VGKFGLE............EEVERLQRDKNVLMQELVRLRQQQQVTEHHLQNVGQKVHVM
HFA6B VGRYGLD............GEMDSLRRDKQVLMMELVRLRQQQQSTKMYLTLIEEKLKKT
HSFA2 VGQYGFD............GEVERLKRDHGVLVAEVVRLRQQQHSSKSQVAAMEQRLLVT
HSFA3 GSPTEVG............GEIEKLRKERRALMEEMVELQQQSRGTARHVDTVNQRLKAA
HFA7A ....................ACNELRREKQVLMMEIVSLRQQQQTTKSYIKAMEQRIEGT
HSFA8 ..KSGLW............KEVDILKGDKQVLAQELIKVRQYQEVTDTKMLHLEDRVQGM
HSFA5 QERAVLQ............EQMDKLSREKAAIEAKLLKFKQQKVVAKHQFEEMTEHVDDM
HFA6A .....LE............GEIHELRRDRMALEVELVRLRRKQESVKTYLHLMEEKLKVT
HFA7B .....PG............VELPQLREERHVLMMEISTLRQEEQRARGYVQAMEQRINGA
HFA4A SERVRMN............NQIERLTKEKEGLLEELHKQDEEREVFEMQVKELKERLQHM
HSFC1 .GMYG...................QDLEDGEIVREIERLKEEQRELEAEIQRMNRRIEAT
HSFA9 ...................TEVESLKEEQSPMRLEMLKLKQQQEESQHQMVTVQEKIHGV
HFA4C SERRSME............DQIERLKNEKEGLLAELQNQEQERKEFELQVTTLKDRLQHM
HSFB4 IDTAAQ........VTALSEDNERLRRSNTVLMSELAHMKKLY...NDIIYFVQNHV...
HSFB1 KNPGSVE.....NMVADLSGENEKLKRENNNLSSELAAAKKQR...DELVTFLTGHLKVR
HSFB3 SS..........FVYTALLDENKCLKNENELLSCELGKTKKKC...KQLMELVERYRGED
HFB2B VGGGSLQRTTSCTTAPELVEENERLRKDNERLRKEMTKLKGLY...ANIYTLMANFTPGQ
HFB2A TGNGGL........SVELLEENEKLRSQNIQLNRELTQMKSIC...DNIYSLMSNYVGSQ
```

HR-B

```
          210       220       230       240       250
HFA1D ENRQQQLMSFLAKAVQSPHFLSQFLQQQNQQNESNRRISDTSKKR.RFKRDGIV....RN
HFA1A EQRQQQIMSFLAKAVQNPTFLSQFIQKQT...DSNMHVTEANKKR.RLREDSTAATESNS
HFA1B EQRQQQMMSFLAKAVQSPGFLNQLVQQNN..NDGNRQIPGSNKKR.RLPVDEQE....NR
HFA1E EQRQQQMMSFLAKAVQSPGFLNQFSQQSN...EANQHISESNKKR.RLPVEDQM....NS
HFA6B ESKQKQMMSFLARAMQNPDFIQQL.VEQK..........EKRKEI.EEAISKKRQRPIDQ
HSFA2 EKRQQQMMTFLAKALNNPNFVQQFAVMSK..........EKKSLF.GLDVGRKRR.....
HSFA3 EQRQKQLLSFLAKLFQNRGFLERLKNFKGK..EKGGALGLEKARK.KFIKHHQPQDSPT
HFA7A ERKQRQMMSFLARAMQSPSFLHQL...............LKQ......RDKKIKE.....
HSFA8 EESQQEMLSFLVMVMKNPSLLVQL.LQPK..........EKNTW....RKAGEGAKIVEE
HSFA5 ENRQKKLLNFLETAIRNPTFVKNFGKKVEQ.....LDISAYNKKR.RLPEVE..QSKPPS
HFA6A EVKQEMMMNFLKKIKKPSFLQSL................RKRNLQ.GIKNREQKQEVISS
HFA7B EKKQRHMMSFLRRAVENPSLLQQI...............FEQK.....RDREEAA.....
HFA4A EKRQKTMVSFVSQVLEKPGLALNLSPCVPE........TNER.KR.RFPRIEFFPDEPML
HSFC1 EKRPEQMMAFLYKVVEDPDLLPRMMLEKER.TKQQQQVSDKKKRR.......VTMSTVKS
HSFA9 DTEQQHMLSFFAKLAKDQRFVERLVKKRKMKIQRELEAAEFVKKLKLLQDQETQKNLLDV
HFA4C EQHQKSIVAYVSQVLGKPGLSLNL.....E........NHERRKR.RF............
HSFB4 ...KPVAPS......NNSSYLSSFLQKQQQ..QQPPTLDYYNTATVNATNLNALNSSPPT
HSFB1 PEQIDKMIK......GGKFKPVESDEESEC..EG........................CD
HSFB3 EDATD.................ESDDEED...............................
HFB2B EDCAHLLPE......G...KPLDLLPERQE..MS.................EAIMASEIE
HFB2A PTDRSYSPG......GSSSQPMEFLPAKR..........................FSEME
```

```
         260        270       280             290       300       310
HFA1D NDSATP..DGQIVKYQPPMHEQAKAMFKQLMKM......EPYKTGDDGFLLGNGTSTTEG
HFA1A HSHSLEASDGQIVKYQPLRND...SMMWNMMKT............DDKYPFLDGFSSPN.
HFA1B GDNVANGLNRQIVRYQPSINEAAQNMLRQFLNTSTSPRYESVSNNPDSFLLGDVPSSTS.
HFA1E GSHGVNGLSRQIVRYQSSMNDATNTMLQQIQQMSNAPSHESLSSNNGSFLLGDVPNSNI.
HFA6B GKRNVE..............DYGDESGYGNDVAASS........................
HSFA2 .................................LTS........................
HSFA3 GGEVVKYEADDWERLLMYDEETENTKGLGGMTSSDPKG......KNLMYPSEEEMSKPD.
HFA7A ....LE..............D.......................................
HSFA8 ....VT..............DEGESNSYGLPLVTYQ........................
HSFA5 EDSH...LDNSSGSSRRESGNIFHQNFSNKLRLELSPA......DSDMNMVSHSIQSSN.
HFA6A ..HGVE..............D.......................................
HFA7B ....MI..............D.......................................
HFA4A EENKTCVVVREEGSTSPSSHTREHQ..VEQLESSIAIW......E...NLVSDSCESML.
HSFC1 EEEEVEEDEGRVFRVMSSSTP.SPSSTENLYRNHSPDGWIVPMTQGQFGSYETGLVAKSM
HSFA9 EREFMAMAATEHNPEPDILVNNQSGNTRCQLNSEDLLVDGGSMDVNGRIEIE........
HFA4C ..........QENSLPPSSSHIE.Q..VEKLESSLTFW......E...NLVSESCEKSG.
HSFB4 SQSSITVLEDDHTNHHDQSNMRKTKLFGVSLPSSKKRSH............HF.......
HSFB1 GGGGAEEGVGEGL...........KLFGVWLKGERKKRD............RD.......
HSFB3 ..........EGL...........KLFGVKLE............................
HFB2B TGIGLKLGED...........LTPRLFGVSIGVKRARRE............EELGAAEEE
HFB2A ....IEEEEE...........ASPRLFGVPIGLKRTRSE............GV.......
```

```
              320       330        340           350       360
HFA1D TEMETSSNQVSGITLKEMPTASEIQSS.SPIETTPENV....SAASEATENCIPSPDDLT
HFA1A ........QVSGVTLQEVLPITSGQSQ.AYASVPSGQP....LSYLPSTSTSLPD....T
HFA1B VDNGNPSSRVSGVTLAEFS..PNTVQS.A........T....NQVPEA..SLAHHPQAGL
HFA1E SDNGSSSNGSPEVTLADVSSIPAGFYP.AMKYHEPCET....NQVMET..NLPFS.QGDL
HFA6B ..................SALIGMSQE.YTYGNMSEFE....MSELDKLA..........
HSFA2 ..................TPSLGTMEE.NLLHDQ.EFD....RMKDDMEM..........
HSFA3 YLMSFPSPE..GLIKQEETTWSMGFDT.TIPSFSNTDAWGNTMDYNDVSEFGFAAETTSD
HFA7A .........................NE.SAKRKRGSSS....MSELEVLA..........
HSFA8 ..................PPSDNNGTA.KSNSNDVNDF....LRNADMLKFCLDENHVPL
HSFA5 EEGASPKGILSGGDPNTTLTKREGLPF.APEALELADTGTCPRRLLLNDNTRVETLQQRL
HFA6A .........................NG.KFVKAEPEEY....GDDIDDQC..........
HFA7B .........................QA.GLIKMEEVEH....LSELEALA..........
HFA4A QSRSM.....MTLDVDESSTFPESPPL.SCIQLSV.DS.............RLKSPPSPR
HSFC1 LSNSTSSTSSSLTSTFSLPESVNGGGG.GGCGSIQGER....RYKETATFGGVVESNPPT
HSFA9 ............................................................
HFA4C LQSSS.....MDHDAAESS.............LSIGDT.............RPKSSK...
HSFB4 .SDQTSKTRLVLDQSDLALNLMTASTR.................................
HSFB1 ....EKNYVVSGSRMTEIKNVDFHAPLWKSSKVCN.........................
HSFB3 ............................................................
HFB2B DDDRREAAAQEGEQSSDVKAEPMEENN.SGNHNGSWLELGK...................
HFB2A ...QVKTTAVVGENSDEETPWLRHYNR.TNQRVCN.........................
```

```
          370          380       390           400        410
HFA1D LPDFT...HMLPENNSEKPPESFMEPNLGG....SSPLLDPDLLIDD.SLSFDI.DDFPM
HFA1A IMPET...SQIPQLTRESINDFPTENFMDTEKNVPEAFISPSPFLDGGSVPIQL.EGIPE
HFA1B VQPNI...GQSPAQGAAPADSWSPEFDLVGCETDSGECFDPIMAVLD...ESEG.DAISP
HFA1E LPPT.........QGAAASGSSSS..DLVGCETDNGECLDPIMAVLDGALELEA.DTL..
HFA6B .............MHIQGLGD.........NSSAREEVLNVEKGNDEEEVEDQQ.Q....
HSFA2 .............LFAAAIDD.........EAN......NSMPTKEEQCLEAMN.V....
HSFA3 GLPDVCWEQFAAGITETGFNWPTGDDDDNTPMNDP.........................
HFA7A .............LEMQGHGK.........QRNMLEE..............EDH.Q....
HSFA8 IIPDLYDDGAWEKLLLLSPSR.........KKTKKQEN.IVKKGKDDLTLEEEE.EDGTM
HSFA5 TSSEETD....GSFSCHLNLTLASAPLPDKTASQIAKTTLKSQELNFNSIETSASEKNRG
HFA6A .............GGVFDYGD.........ELHIASM..............E.H.Q....
HFA7B .............LEMQGYGR.........QRTD..........................
HFA4A IIDMNCE....PDGSKEQNTVAAPPP..................................
HSFC1 TPPYPF......SLFRGGF.........................................
HSFA9 ............................................................
HFA4C .IDMNSE....PP.......VTVTAP..................................
HSFB4 ............................................................
HSFB1 ............................................................
HSFB3 ............................................................
HFB2B ............................................................
HFB2A ............................................................
```

```
                    420       430       440       450
HFA1D  DSD............IDPVDYGLLERLLM.SSPV...PDNMDSTPVDN...ETEQEQ...
HFA1A  DPE..IDELMS........NFEFLEEYMP.ESPVFG....DATTLENNNNNNNNNNNNNN
HFA1B  EGEGKMNELLEGVPKLPGIQDPFWEQFFSVELPAIADTDDILSGSVENNDLVLEQEP...
HFA1E  ......NELL......PEVQDSFWEQFIG.ESPVIGETDELISGSVEN.ELILEQLELQS
HFA6B  .........GYHKENNEIYGEGFWEDLLN..............................E
HSFA2  .........MMRDGNLEAALDVKVEDLV................................
HSFA3  ............................................................
HFA7A  .........LVVERELD...DGFWEELL................................
HSFA8  ELD.KSYMLKLISEEMEKPDDFEFGQLT................................
HSFA5  RQEIAVGGSQANAAPPARVNDVFWEQFLT.ERPGSS...DNEEASSTYRGNPYEEQEEK.
HFA6A  .........GQGEDEIEMDSEGIWKGFV................................
HFA7B  ..........GVERELD...DGFWEELL................................
HFA4A  .............PPVAGANDGFWQQFFS.ENPGST...EQREVQLERKD...DKDKAGV
HSFC1  ............................................................
HSFA9  ............................................................
HFA4C  .............APKTGVNDDFWEQCLT.ENPGST...EQQEVQSERRDVGNDNNGNKI
HSFB4  ............................................................
HSFB1  ............................................................
HSFB3  ............................................................
HFB2B  ............................................................
HFB2A  ............................................................
```

```
             460       470       480
HFA1D  ...NGWDKT.KHMDNLTQQMGLLSPETLDLSRQNP
HFA1A  NNNNNNNTNGRHMDKLIEELGLLTSETEH......
HFA1B  ...NEWTRNEQQMKYLTEQMGLLSSEAQRK.....
HFA1E  TLSNVWSKN.QQMNHLTEQMGLLTSDALRK.....
HFA6B  GQNFDFEGDQENVDVLIQQLGYLGSSSHTN.....
HSFA2  GSPLDW..DSQDLHDMVDQMGFLGSEP........
HSFA3  ...................................
HFA7A  ........SDESLASTS..................
HSFA8  ......PERSRNLEILTEQMELLASNE........
HSFA5  RNGSMMLRNTKNIEQLTL.................
HFA6A  ...L....SEEEMCDLVEHFI..............
HFA7B  ...MNNENSDEEEANVKQD................
HFA4A  RTEKC.WWNSRNVNAITEQLGHLTSSERS......
HSFC1  ...................................
HSFA9  ...................................
HFA4C  GNQRTYWWNSGNVNNITEKAS..............
HSFB4  ...................................
HSFB1  ...................................
HSFB3  ...................................
HFB2B  ...................................
HFB2A  ...................................
```

Figure S4. Sequence alignment of Heat shock proteins of *Arabidopsis thaliana.* Multiple sequence alignment was constructed using full length sequences using BLAST. The alignment was depicted using Espript v.3. The AHA motif is highlighted in light pink. DNA binding domain (grey), HR-A region (blue), HR-B region (orange) and HR-A/B domain (pink) are indicated on the top of the alignment by continuous lines.

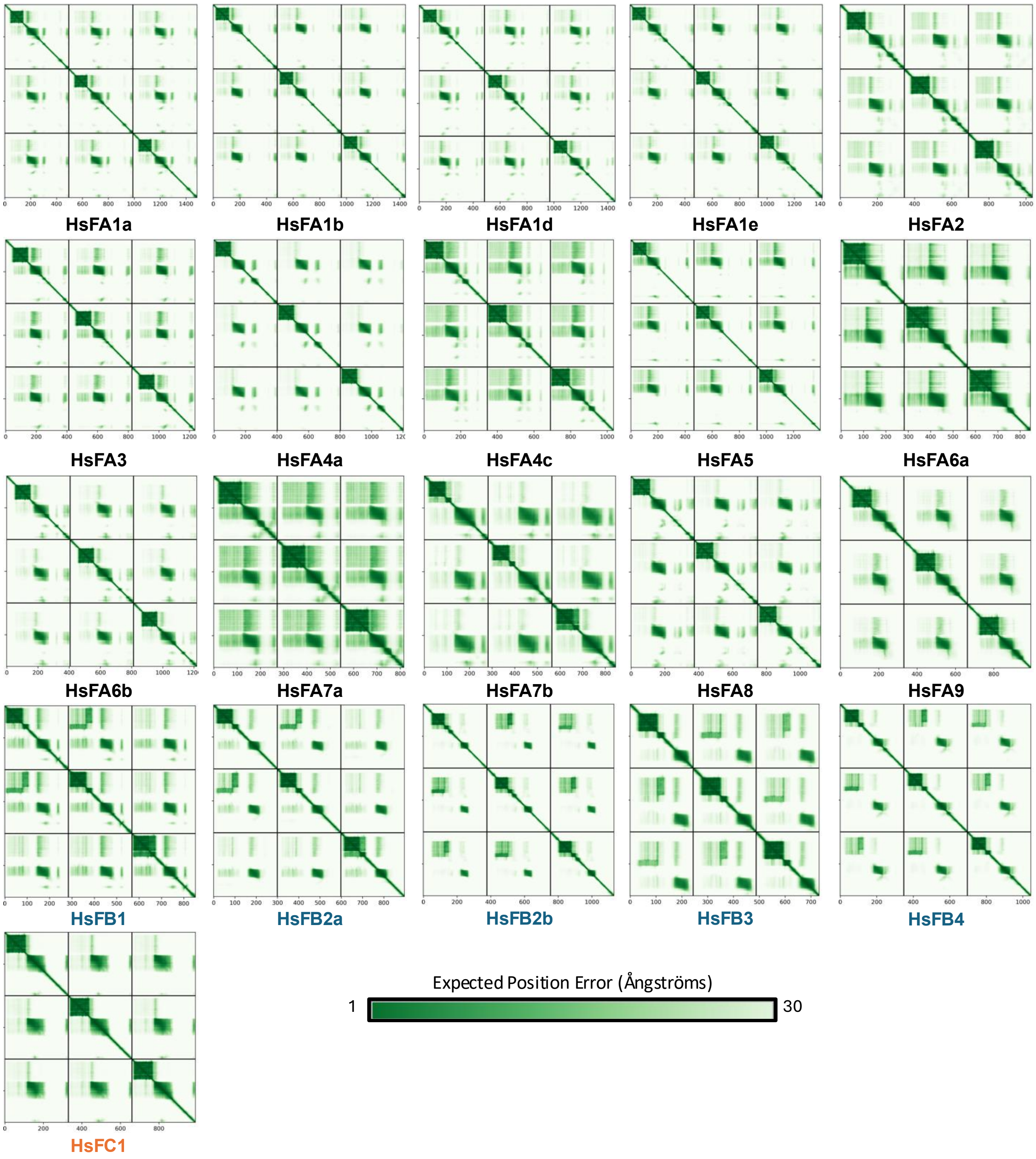

Figure S5. Predicted aligned error plot (PAE) of 21 homo-trimeric heat shock proteins from *Arabidopsis thaliana*. The PAE diagram obtained by AlphaFold, highlights confidence in specific protein-protein interactions.

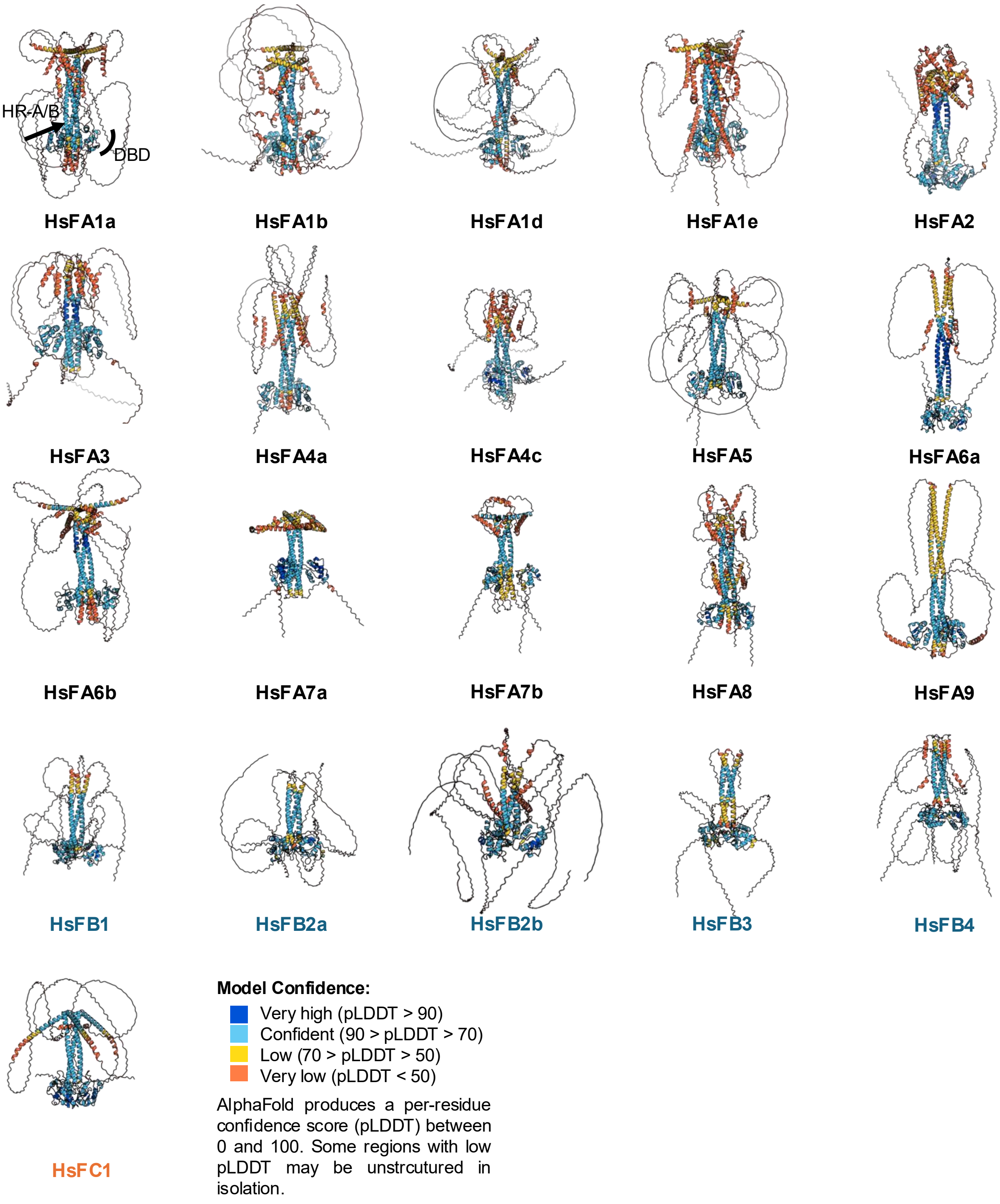

Figure S6. Predicted three-dimensional structures of 21 homo-trimeric heat shock proteins from *Arabidopsis thaliana*. Proteins are represented as homo-trimers. The level of confidence for proteins is represented by their pLDDT values as determined by AlphaFold: very high above 90 (blue), confident between 70 and 90 (pale blue), low between 50 and 70 (yellow) and very low below 50 (orange).

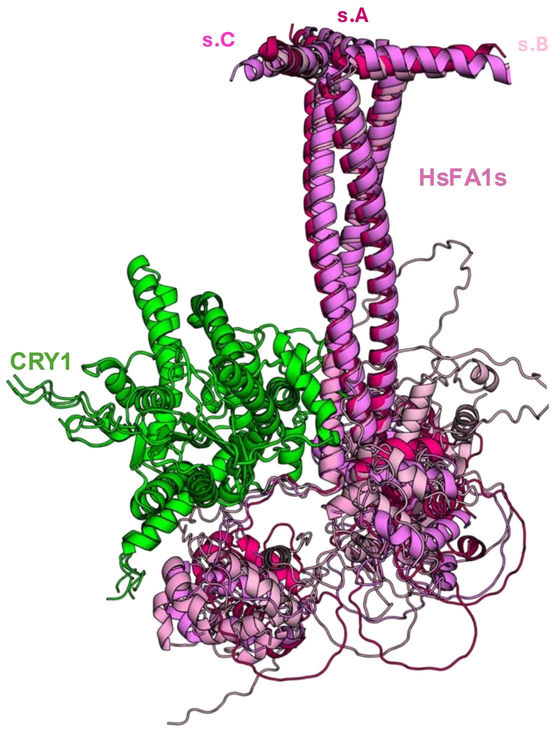

Figure S7. CRY1 interacts with HsFA1s. Structural alignments of CRY1-HsFA1s predicted by AlphaFold. Monomeric CRY1 is represented in pale green and the HsFA1s trimer in pink. HsFA1s are colored in pink tone. HsFA1a (purple), HsFA1b (light pink), HsFA1e (hot pink), HsFA1d (pink). Disordered predicted residues are omitted for clarity.

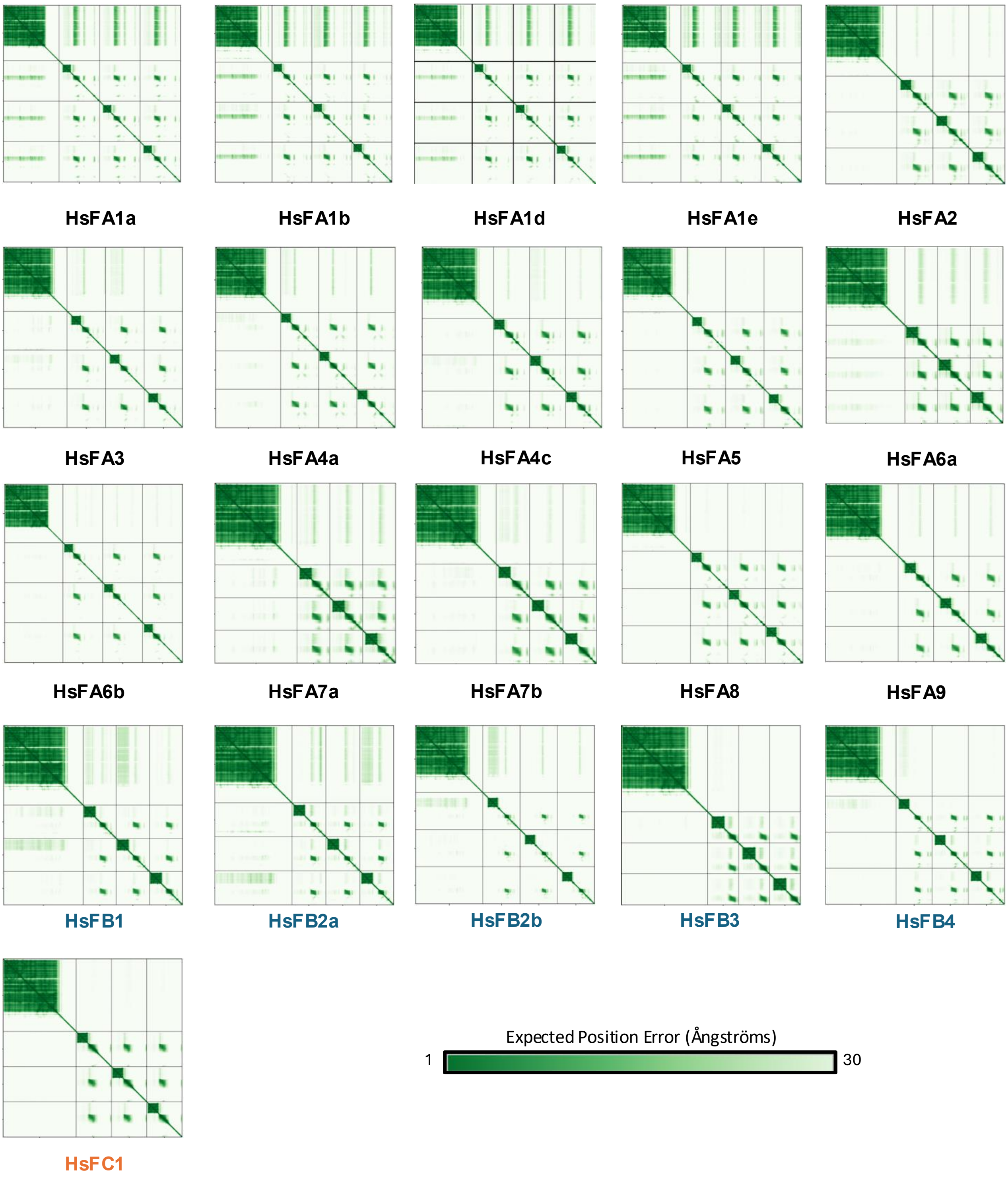

Figure S8. Predicted aligned error plot (PAE) of 21 CRY1-HsFs interaction from *Arabidopsis thaliana*. The PAE diagram obtained by AlphaFold, highlights confidence specific protein-protein interactions.

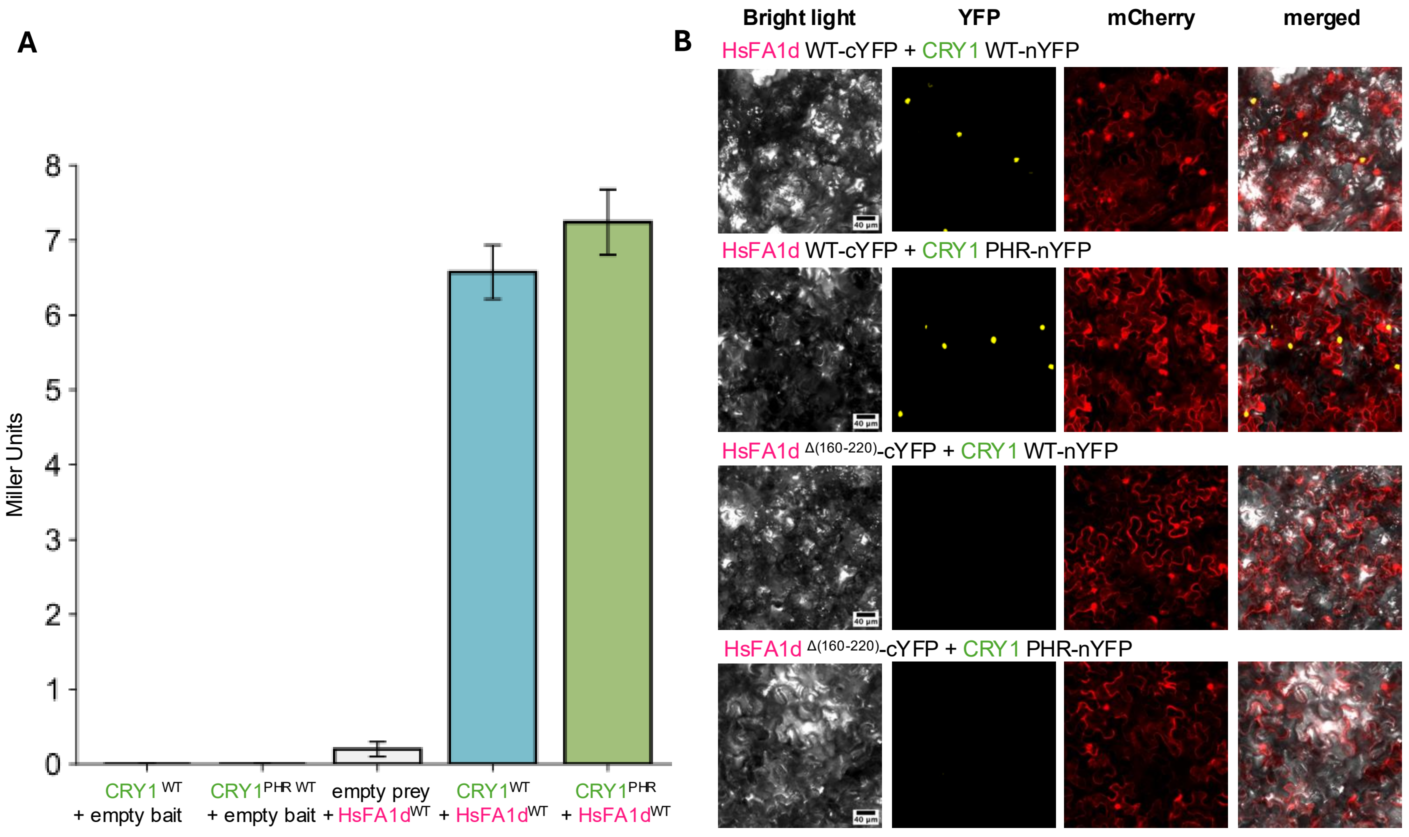

Figure S9: The CRY1 PHR domain interacts with the HsFA1d HR-A region. (A) Yeast 2-hybrid CRY1-HsFA1d mutants. This assay shows interaction between pGADT7 (AD), empty prey, CRY1^{WT}, CRY1$^{WT\ PHR}$ and pGBKT7 (BD), empty bait, HsFA1d^{WT}. Bars show mean ± Standard deviation (n=3). **(B)** Bimolecular fluorescence complementation assay between CRY1 and HsFA1d in *N.benthamiana* mesophyll. This assay shows interaction between CRY1^{WT}-nYFP, CRY1$^{PHR\ WT}$-nYFP and HsFA1d^{WT}-cYFP or HsFA1d$^{\Delta(160\text{-}220)}$-cYFP.

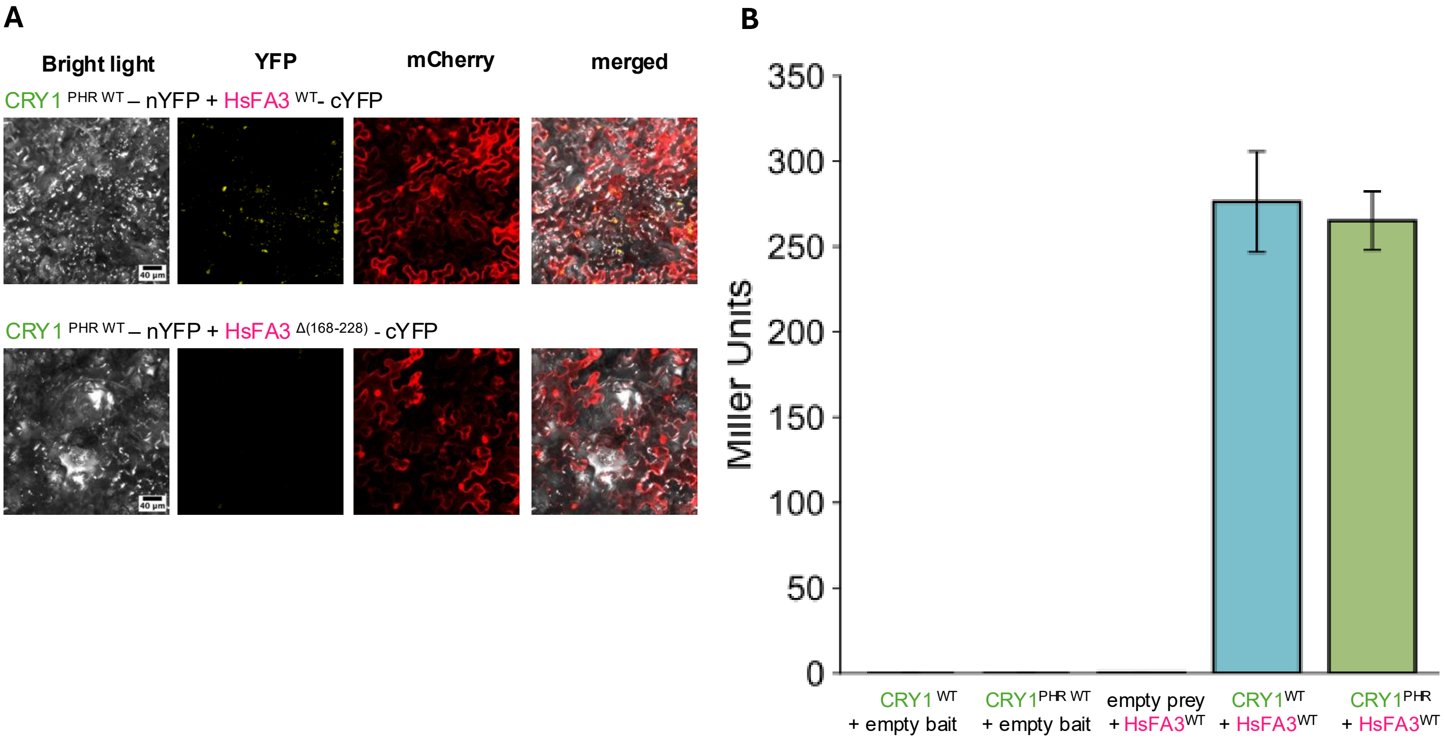

Figure S10: CRY1 PHR domain interacts with HsFA3. Yeast 2-hybrid CRY1-HsFA3. This assay shows interaction between pGADT7 (AD), empty prey, CRY1^{WT}, CRY1$^{PHR\ WT}$ and pGBKT7 (BD), empty bait, HsFA3^{WT} Bars show mean ± Standard deviation (n=3).

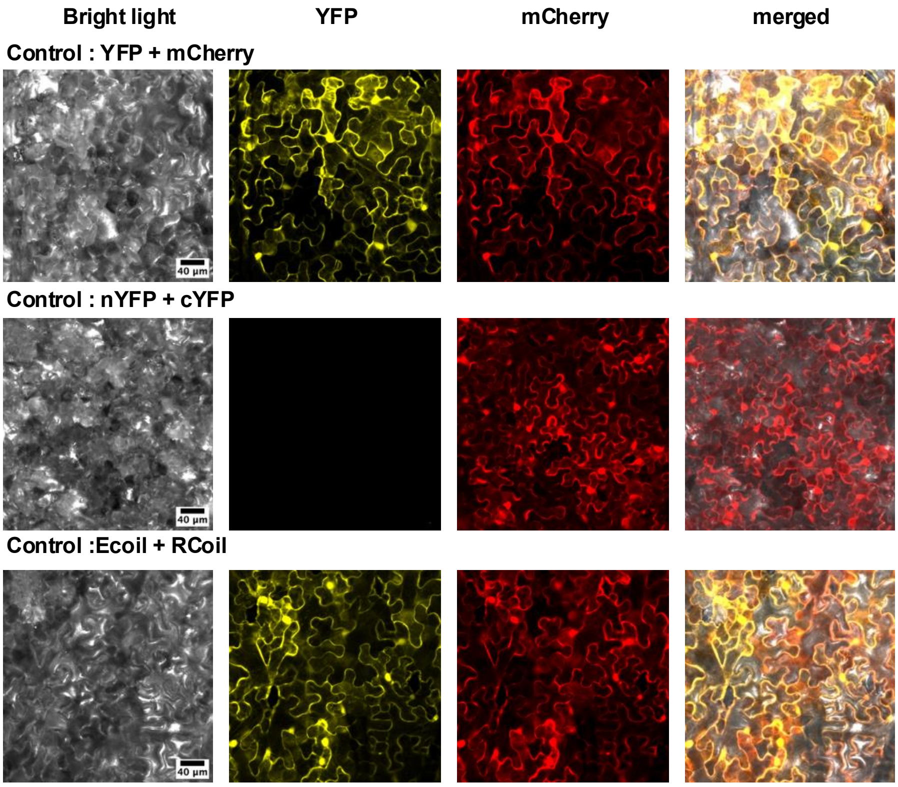

Figure S11. Bimolecular fluorescence complementation assay controls used in this study. The full-length YFP and mCherry were used as positive constitutive controls. Empty vectors nYFP and cYFP were used as negative auto complementation controls. Ecoil and Rcoil are known to interact, the proteins were fused to nYFP and cYFP respectively and used as positive controls to validate the YFP split.

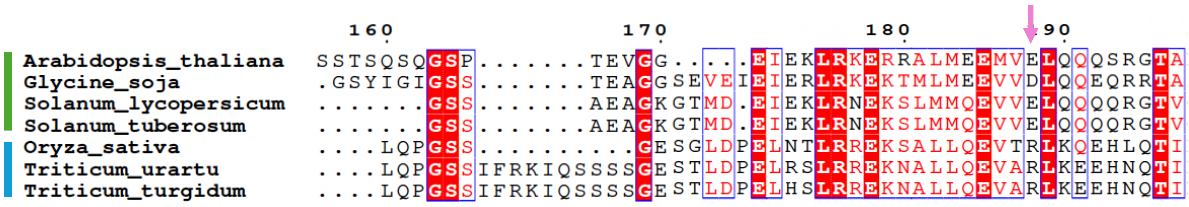

Figure S12. Sequence alignment of Heat shock proteins from common crops. Multiple sequence alignment of HsFA3 proteins from common crops species compared to *Arabidospsis thaliana* HsFA3. The alignment focuses on the HR-A region. The position of E188 in Arabidopsis HsFA3 is indicated with a pink arrow. Dicotyledonous sequences are grouped and highlighted in green, whereas monocotyledonous sequences are shown in blue.

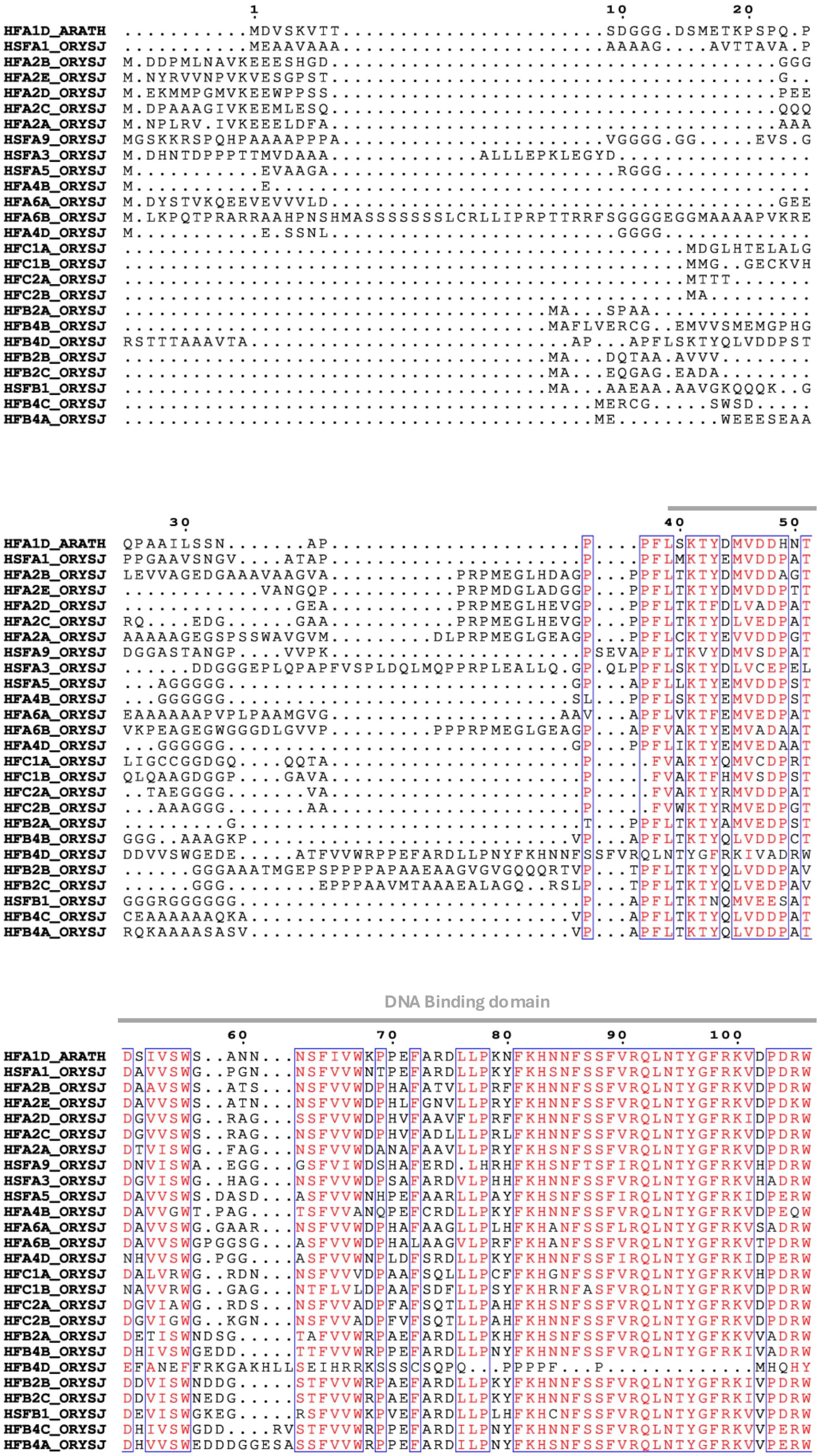
DNA Binding domain

```
              110       120       130
HFA1D_ARATH  EFANEGFLRGQKHLLQSITRRKPA.....H............................GQ
HSFA1_ORYSJ  EFANEGFLRGQKHLLKTINRRKPT.....H............................GN
HFA2B_ORYSJ  EFANENFLRGQRHLLKNIKRRKPPS..................................H
HFA2E_ORYSJ  EFANEGFLRGQKHLLKSIKRRKPPN..................................S
HFA2D_ORYSJ  EFANDGFLRGQRHLLKMIKRRRPLS..................................Y
HFA2C_ORYSJ  EFANEGFLRGQRHLLKTIKRRKPPS..................................N
HFA2A_ORYSJ  EFANEGFLRGKKELLKTIKRRRPPP..................................S
HSFA9_ORYSJ  EWANEGFIMGQKHLLKTIKRRKKS.....S............................QE
HSFA3_ORYSJ  EFAHEDFLRHSKHLLKKIVRRRSSP..................................T
HSFA5_ORYSJ  EFANEYFIKGQKHLLKNIHRRKPIH..................................S
HFA4B_ORYSJ  EFANEDFIKGQRHRLKNIHRRKPIF..................................S
HFA6A_ORYSJ  EFANEDFLGGQRHLLANIRRRRRGA..................................G
HFA6B_ORYSJ  EFANEAFLAGQKHLLKNIKRRRVSK..................................P
HFA4D_ORYSJ  EFANEDFIRGHTHLLKNIHRRKPVH..................................S
HFC1A_ORYSJ  EFAHESFLRGQTHLLPRIVRRKKR....GE............................GG
HFC1B_ORYSJ  EFAHESFLRGQAQLLPRIVRKKKK....G.............................GA
HFC2A_ORYSJ  EFAHVSFLRGQTHLLRRIVRRSS....................................G
HFC2B_ORYSJ  EFAHASFLRGQTHLLRNIVRRGSAAAGGGG............................GG
HFB2A_ORYSJ  EFANDCFRRGEKHLLGGIQRRKGS............GT...........GGAGAAPAGG.
HFB4B_ORYSJ  EFANEFFRKGAKHLLAEIHRRKSS...QP....PPPPM...PHQP..........YHHHH
HFB4D_ORYSJ  ............PLSLFSPPTTPRSPPVGAAAAAAYH....FQEE..........YCSSP
HFB2B_ORYSJ  EFANDCFRRGERRLLCEIHRRKVT........PPAPAA...........TTAAVAAA...
HFB2C_ORYSJ  EFANDCFRRGEKRLLCDIHRRKVVAAAAAAPPPPSPGM...........ATAAAAVASGA
HSFB1_ORYSJ  EFANGNFRRGEQGLLSGIRRRKAT.............T...........PQSSKSCGSG.
HFB4C_ORYSJ  EFANEFFRKGEKQLLTEIHRRKTSSASTAS..PSPPPFFAPPHFPLFHHPGVAAAQHHHA
HFB4A_ORYSJ  EFANEFFRKGEKQLLCEIHRRKSAAATWPPFPPPPPPPFFAPRHF.......AAGAFFRHG
```

```
                                                  140       150
HFA1D_ARATH  ................................GQGHQRSQHSNGQNSS.....VSACVEV
HSFA1_ORYSJ  ................................NQVQQPQLPA....AP.....VPACVEV
HFA2B_ORYSJ  ................................TASN..........QQ....SLGPYLEV
HFA2E_ORYSJ  ................................SPS...........QQ....SLGSFLEV
HFA2D_ORYSJ  ................................LPGS..........QQ....ALGTCLEV
HFA2C_ORYSJ  ................................APPS..........QQ...QSLTSCLEV
HFA2A_ORYSJ  ................................SPPSSSSSSSSSQHQQ...QPAAACLEV
HSFA9_ORYSJ  ................................SPSEIQKAPV..KTAP.....GTENIEI
HSFA3_ORYSJ  ................................QQSGLQPGSS..................
HSFA5_ORYSJ  ................................HS...HPP..............GALPDN
HFA4B_ORYSJ  ................................HSSHSQGA..............GPLTDN
HFA6A_ORYSJ  ................................TGSTTPRA.................VNC
HFA6B_ORYSJ  ................................LVDSQLRNK..............ASVVF
HFA4D_ORYSJ  ................................HSLQNQIN..............GPLAES
HFC1A_ORYSJ  ................................GGGGGASCSFGGGAGEH..QVAAAAASV
HFC1B_ORYSJ  ................................APGCRELCE...................
HFC2A_ORYSJ  ................................GGGAKRKEEAGGCG..............
HFC2B_ORYSJ  ................................GGGKRRDASADGGG..............
HFB2A_ORYSJ  ................IPTAIPIS.....SPP........TSSGGE.....PAVSS..SP
HFB4B_ORYSJ  ............HLNPFSLPPPP..........PAYHHHHLIQEEPA.....TTAHCTVA
HFB4D_ORYSJ  ADYAGGGGDLL....AALSEDNRQLRRRNSLLLSE..............LAHMRKLYNDI
HFB2B_ORYSJ  ................IPMALPVTTTRDGSPVLSGEEQVISSSSSP....EPPLVLPQAP
HFB2C_ORYSJ  VT....VAA.....APIPMALPVT..RAGSPAHSSEEQVLSSNSGSGEEHRQASGSGSAP
HSFB1_ORYSJ  ................VNVAFPPP.....LPPLPPEPSATTSSGND.....RSSSSASSP
HFB4C_ORYSJ  FVGDDGVVAAHG....IGMPFPQPHWREPN.LPVA..TRLLALGGPA.....PS..PSSA
HFB4A_ORYSJ  ....DGML..HGRLGALVTTTERRHWFESAALPVAPSSRLLSQLGPV.....IA..PARR
```

HR-A

HR-A/B domain

```
              160       170       180       190       200       210
HFA1D_ARATH  GKFG.LEEEVE.....RLKRDKNVLMQELVRLRQQQQSTDNQLQTMVQRLQGMENRQQQL
HSFA1_ORYSJ  GKFG.MEEEIE.....MLKRDKNVLMQELVRLRQQQQTTDHQLQTLGKRLQGMEQRQQQM
HFA2B_ORYSJ  GHFG.YDAEID.....RLKRDKQLLMAEVVKLRQEQQNTKANLKAMEDRLQGTEQRQQQM
HFA2E_ORYSJ  GHFG.YEGEID.....QLKRDKHLLMAEVVKLRQEQQNTKSDLQAMEQKLQGTEQKQQHM
HFA2D_ORYSJ  GQFG.LDEEID.....RLKRDKNILLAEVVKLRHKQQSTKANMRAMEERLQHAEQKQVQM
HFA2C_ORYSJ  GEFG.FEEEID.....RLKRDKNILITEVVKLRQEQQATKDHVKAMEDRLRAAEQKQVQM
HFA2A_ORYSJ  GQFG.RDGVVN.....RLQRDKSVLIAEVVKLRQEQQTTRAQMQAMEERISAAEQKQQQM
HSFA9_ORYSJ  GKYGGLEKEVE.....TLKRDKALLMQQLVDLRHYQQTSNLEVQNLIERLQVMEQNQQQM
HSFA3_ORYSJ  GESG.LDPELN.....TLRREKSALLQEVTRLKQEHLQTIEQMSTLNQRLESAEDRQKQM
HSFA5_ORYSJ  ERAI.FEDEIE.....RLSREKSNLQADLWKSKQQQSGTMNQIEDLERRVLGMEQRQTKM
HFA4B_ORYSJ  ERKD.YEEEIE.....RLKSDNAALSSELQNNTLKKLNMEKRMQALEEKLFVVEDQQRSL
HFA6A_ORYSJ  GGGG.GEGEVE.....RLRRDKEALARELARLRRQQQEARAQLLDMERRVRGTERRQEQC
HFA6B_ORYSJ  GQPE.APGEVV.....SLKRDRAALRAEVIMLKQQYNACKSQLIAMEEMVRNIERRQQQT
HFA4D_ORYSJ  ERRE.LEEEIN.....RLKYEKSILVADLQRQNQQQYVINWQMQAMEGRLVAMEQRQKNI
HFC1A_ORYSJ  GMSGEEEDAAE.....DVLAKEAALFEEVQRLRHEQTAIGEELARMSQRLQATERRPDQL
HFC1B_ORYSJ  ..EGEEVRGT...............IEAVQRLREEQRGMEEELQAMDQRLRAAESRPGQM
HFC2A_ORYSJ  ...GGGEAAAG.....DVDEESAVVALEVARLRREQREIEGRVAAMWRRVQETERRPKQM
HFC2B_ORYSJ  ...GGG............DEDMTMVATEVVRLKQEQRTIDDRVAAMWRRVQETERRPKQM
HFB2A_ORYSJ  PRGAAGIAAGVSGAVAELEEENARLRRENARLARE..............LARARRVCDGV
HFB4B_ORYSJ  GD.GGEGGDFL....AALSEDNRQLRRRNSLLLSE..............LAHMKKLYNDI
HFB4D_ORYSJ  IYFLQNHV..........................................EP........
HFB2B_ORYSJ  SGSGSGGVAS.....GDVGDENERLRRENAQLARE..............LSQMRKLCNNI
HFB2C_ORYSJ  GGGGGGSASG.....GDMGEENERLRRENARLTRE..............LGHMKKLCNNI
HSFB1_ORYSJ  PR.............ADITSENEQLRKDNQTLTME..............LARARRHCEEL
HFB4C_ORYSJ  EAGGAGRAATA....AVLMEENERLRRSNTALLQE..............LAHMRKLYNDI
HFB4A_ORYSJ  AAATPEEEA........LMQENHRLLRGNAALVQE..............LAHMRKLYSDI
```

```
                      HR-B
                220          230          240          250          260
HFA1D_ARATH  MSFLAKAV.QSPHFLSQFLQQQNQQNE.SNRRISDTSKKRRF.K.RDGIVRN.....ND.
HSFA1_ORYSJ  MSFLAKAM.HSPGFLAQFVQQNEN....SRRRIVASNKKRRLPK.QDGSL.D.....SE.
HFA2B_ORYSJ  MAFLARVM.KNPEFLKQLMSQNE.....MRKELQDAISKKRRRRIDQGPEVD........
HFA2E_ORYSJ  MAFLSRVM.HNPEFIRQLFSQSE.....MRKELEEFVSKKRRRRIDQGPELD........
HFA2D_ORYSJ  MGFLARAM.QNPDFFHQLIHQQD.....KMKGLEDTFSKKRTRSIDIVPFLN........
HFA2C_ORYSJ  MGFLARAM.RNPEFFQQLAQQKE.....KRKELEDAISKKRRRPIDNVPFYD........
HFA2A_ORYSJ  TVFLARAM.KNPGFLQMLVDRQAGQHGARNRVLEDALSKKRRRPIEYLLTRN........
HSFA9_ORYSJ  MALLAIVV.QNPSFLNQLVQQQQQQRR.SNWWSPDGSKKRRFHALEQGPVTD.....QE.
HSFA3_ORYSJ  VSFLAKLL.QNPTFLRQLKMHRQQ......KEIDSTRVKRKFLKHVPHGNID........
HSFA5_ORYSJ  IAFLQQAS.KNPQFVNKLVKMAEASSI.....FTDAFNKKRRLPGLDYSIEN....TETT
HFA4B_ORYSJ  ISYVREIV.KAPGFLSSFVQQQ............DHHRKKRRLPIPISFHED.ANTQENQ
HFA6A_ORYSJ  TEFLARAL.RSPDVLDNIARRHAA...........AVERKKRRMLAAAADDD........
HFA6B_ORYSJ  IGFFAKVL.TNPAFVQQVLLNYVN.....KNGLRGAA..KRQRLMENEEQHA........
HFA4D_ORYSJ  VASLCEMLQRRGGAVSSSLLES............DHFSKKRRVPKMDLFVDDCAAGEEQK
HFC1A_ORYSJ  MSFLAKLA.DDPNAVTGHLLEQAAERK.RRRQHLPSHEPTVCPLPPAPPPQP.....PQP
HFC1B_ORYSJ  MAFLAKLA.DEPGVVLRAMLAKKEE...................................
HFC2A_ORYSJ  LAFLVKVV.GDPQVLRRLVDRDN.......TN............................
HFC2B_ORYSJ  LAFLLKVV.GDRDKLHRLVGGGGNGNG.AATA............................
HFB2A_ORYSJ  RRLVSRYD..........................................HD........
HFB4B_ORYSJ  IYFLQNHV..........................................AP........
HFB4D_ORYSJ  ............................................................
HFB2B_ORYSJ  LLLMSKYA..........................................ST........
HFB2C_ORYSJ  LLLMSKYA..........................................AT........
HSFB1_ORYSJ  LGFLSRFL..........................................DV........
HFB4C_ORYSJ  IYFVQNHV..........................................RP........
HFB4A_ORYSJ  IYFVQNHV..........................................RP........

                   270          280          290          300
HFA1D_ARATH  ...SATPDGQIVKY..QPPMHEQAKAMFKQLMKMEPYKT.....GDDGFLLG..NGTSTT
HSFA1_ORYSJ  ...SASLDGQIVKY..QPMINEAAKAMLRKILKLDSSHRFESMGNSDNFLLE..NYMPNG
HFA2B_ORYSJ  .....DVGTSSSIEQESPALFDPQESVEFLIDGIPSDLEN....................
HFA2E_ORYSJ  .....SMGTGSSPEQVSQVMFEPHDPVDSLFNGVPSDLES....................
HFA2D_ORYSJ  .....PGEVSQGDQLESTLLFDPRPFAELNDEPAKSELEN....................
HFA2C_ORYSJ  .....PGETSQTEQLDSPYLFDSGVLNELS.EPGIPELEN....................
HFA2A_ORYSJ  .....GETCAAG..........................ES....................
HSFA9_ORYSJ  ...TSGRGAHIVEY..LPP.........................................
HSFA3_ORYSJ  .....SGESSSQHTGESNLDFSPTSLDLPATHSDILDLQNFLLEDGDLN...........
HSFA5_ORYSJ  SFYDDHSSTSKQETGNLL...NQHFSDKLRLGLCPAMTESNIITLSTQSSNE.DNRSPHG
HFA4B_ORYSJ  IMPCDL..TNSPAQ.....TFYRESFDKMESSL..NSLENFLREASEEF.GN.DISYDDG
HFA6A_ORYSJ  .....GLT.............................FEA....................
HFA6B_ORYSJ  .....DSPLNKGMEAASVMEADVSPGSTGC..GTVGKVET....................
HFA4D_ORYSJ  VFQFQGIGTDAPAMPPVLPVTNGEAFDRVELSL..VSLEKLFQRANDACTAA.EEMYSHG
HFC1A_ORYSJ  LLALAGAAAMDGTYWWTTEHHHHHHHQMKPMTVLPSLEPPTASCGVHQVPEL......GG
HFC1B_ORYSJ  .LAAAGNNGSD...............................PCKRRRIGAD......TG
HFC2A_ORYSJ  ..AAASN..ADDS........AVHHQVKRPRLLLDSSSTTTTHGDRHLVTAAADGFYAGG
HFC2B_ORYSJ  ..AAADNGFADAA........RAGCGEKRARLLLDGDNT...................GA
HFB2A_ORYSJ  ............................................................
HFB4B_ORYSJ  ............................................................
HFB4D_ORYSJ  .......................V..............................APPP..
HFB2B_ORYSJ  ............................................................
HFB2C_ORYSJ  ............................................................
HSFB1_ORYSJ  ............................................................
HFB4C_ORYSJ  ............................................................
HFB4A_ORYSJ  ............................................................

            310          320          330          340
HFA1D_ARATH  EGTEMETSSNQV..SGITLKEMPTASE...IQSSSPIE.....TTPE.............
HSFA1_ORYSJ  QGLD.SSSSTRN..SGVTLAEVPANSGLPYVATSSGLSAICSTSTPQIQCPVVLDNGIPK
HFA2B_ORYSJ  ..............SAMDAGG.LVEPQDFDVGASEQQQI.....GPQ.GELND.NFWEEL
HFA2E_ORYSJ  ..............SSVEANG.GKAQQDVASSSSEHGKI.....KPSNGELNE.DFWEDL
HFA2D_ORYSJ  ..............LALNIQGLGKGKQDVNRTRNQPRNQ.....ASNETELTD.DFWEEL
HFA2C_ORYSJ  ..............LAVNIQDLGKGKVDEER.....QNQ.....TNGQAELGD.DFWAEL
HFA2A_ORYSJ  ..............AAMLAAD.GVAEPDGDTT..PRGDG.....GGGGGGDTE.SFWMQL
HSFA9_ORYSJ  .....................VPETSG.....QVNPVE......................
HSFA3_ORYSJ  ..............LAMLPENIGLDGIEAPDDIGALVQG.....FDTQEELEL.GSGVEL
HSFA5_ORYSJ  KHPECDMMG.RE.CLPLVPQMMELSDTGTSICPSKSSCF.....APPISDEGLLTCHLSL
HFA4B_ORYSJ  ......VPG..P.SSTVVLTELHSPGESDPRVSSPPTRM.....RTSSAGAG..DSHS..
HFA6A_ORYSJ  ..............LALAAAA......DTSHST.............GGAVTTD.MIWYEL
HFA6B_ORYSJ  ..............TPMCNFQ.........................NIENMCD.DVWEEL
HFA4D_ORYSJ  ......HGGTEP.STAICPEEMNTAPMETGIDLQLPASL.....HPSSPNTG..NAHLHL
HFC1A_ORYSJ  GGV................MGLTTDGEA......KVE.........PPFPFCLLGQAFF.
HFC1B_ORYSJ  RG..................GVATGGDAAEMAQSRGT.........VPFPFSVLGQVFY.
HFC2A_ORYSJ  CGPEAAAAAAFVPDDAVDFTGLYTGGDGFGNAVVDA.....GVDYPPAYAFPVVDSGY..
HFC2B_ORYSJ  FGP...........DAVDFAGFYTGADMFPDVAVDAAAAAAGGSAGCSFAFGV.DSGY..
HFB2A_ORYSJ  .......................HGGGEEEA.............................
HFB4B_ORYSJ  .......................VTTTTTTPSSTAMA................AAQHH..
HFB4D_ORYSJ  ...........LAAATSCR.........................................
HFB2B_ORYSJ  .......................QQLDAANASSAAGNNNN....NNCSGESAEAATPLPL
HFB2C_ORYSJ  .......................QHVEGS.....AGISSI....ANCSGESSEAVPPPPP
HSFB1_ORYSJ  .......................RQLDLRLL.............................
HFB4C_ORYSJ  .......................VAPSPAAAAFLQGL................GM....Q
HFB4A_ORYSJ  .......................VAPSPAAAAALHGL................GVLRPPP
```

```
                  350         360          370       380
HFA1D_ARATH   ................NVSAAS..EATENCIPSPDDLTL...PDFTHMLPENNSEKPPES
HSFA1_ORYSJ   E.............VPNMSAVP..SVPKAVAPGPTDINILEFPDLQDIVAEENVDIPGGG
HFA2B_ORYSJ   L.............NEG..LVG..EENDNPVV..................E....DDMNV
HFA2E_ORYSJ   L.............HEG..GLD..EDTRNPAI.......................DDMNL
HFA2D_ORYSJ   L.............NE...GAR..DDAGIPGM..................ERRRPRYVDA
HFA2C_ORYSJ   L.............VEDFTGKE..EQSELDG......................KIDGIDE
HFA2A_ORYSJ   L.............SLG...LE..EKQREDGVAGGVQESNS.GGADVDNDEEDDDDDVDV
HSFA9_ORYSJ   ..................GAIC..SANSQPVPSPAVATPMD.......MQTSNVADTLGS
HSFA3_ORYSJ   L.............EIPPASGP..RGQDPTIGRSKGKNVLS.PGLDATSSEA...DCLGS
HSFA5_ORYSJ   T.............LASC..SM..DVDKSQGLNANGT.TID.NPTEAATATMEKDDTIDR
HFA4B_ORYSJ   ....................SR..DVAESTSC.......AE.SP.PIPQMHSRVDTRAK.
HFA6A_ORYSJ   L.............GEEQ..AE..IDIEVDQLVASASAAAD.TASEAEPWEEMGEEEVQE
HFA6B_ORYSJ   D.............ALPETGME..QEEKAGIGSFDVEEFVG.RPCGW.......VDDCPY
HFA4D_ORYSJ   ....................ST..ELTESPGF.......VQ.SP.ELPMAEIREDIHVTR
HFC1A_ORYSJ   ............................................................
HFC1B_ORYSJ   ............................................................
HFC2A_ORYSJ   ............................................................
HFC2B_ORYSJ   ............................................................
HFB2A_ORYSJ   ...........................GEGDVKPMLFGVAI.GGKRSREENGEDEEEEEE
HFB4B_ORYSJ   ...........LPAAASCR.........................................
HFB4D_ORYSJ   ....LVEL.GPSTTERRRCAASPSGDND...DDAA.....VRLFGVRLDDDH......GK
HFB2B_ORYSJ   ..PAVLDLMPSCPGAASAAAP..VSDNEEGMMSAKLFGVSI.GRKRMR........HDG.
HFB2C_ORYSJ   LPPAILDLMPSCPALATAAAAAGLAIDGEPDPSARLFGVSI.GLKRTRDDAAAAADEDG.
HSFB1_ORYSJ   ...........................MQEDMRAAAGGV...GGEQRVQEHAREEKCVKL
HFB4C_ORYSJ   A........RKKPAAANVLNNS....................GGSTTSSSSLTIAEEPS.
HFB4A_ORYSJ   A........GGKAPASEVRGAS....................GRSATSSSSLTVAEDQP.

                 390      400        410               420      430
HFA1D_ARATH   FMEPNLGGSSPLLDPDLLIDDSLSFDID.D....FPMDSD...IDPVDYGLLERLLMSSP
HSFA1_ORYSJ   FEMPGPEGVFSLPEEG...DDSVPIETD.E....ILYNDDTQKLPAIIDSFWEQFLVASP
HFA2B_ORYSJ   LSEKMGYLNSNGPTA...............GE............................
HFA2E_ORYSJ   LSQKMGYLNSSSTKS...............PQ............................
HFA2D_ORYSJ   LAQKLGYLSNSSQK..............................................
HFA2C_ORYSJ   LAQQLGYLSSTSPK..............................................
HFA2A_ORYSJ   LVQSIYHLSPK.................................................
HSFA9_ORYSJ   SEEP.............FADNSTLHEWD.DNDMQLLFDDN...LDPILPPFENDGQMGPP
HSFA3_ORYSJ   FSDNMGMLSDSMLQT...............AGKLMDADDDERIWGVDASSALQSSCSGTS
HSFA5_ORYSJ   ...SFDDNQKKSADSR.....TADATTP.RADARVASEAPAAPAAVVNDKFWEQFLTERP
HFA4B_ORYSJ   .VSEIDVNSEPAVTE..........TGP.SRDQ..PAEEPPAVTPGANDGFWQQFLTEQP
HFA6A_ORYSJ   LVQQIDCLASPSS...............................................
HFA6B_ORYSJ   LVEPMQFVEH..................................................
HFA4D_ORYSJ   YPTQADVNSEIA.SS..........TDT.SQDG..TSETEASHGP.TNDVFWERFLTETP
HFC1A_ORYSJ   ............................................................
HFC1B_ORYSJ   ............................................................
HFC2A_ORYSJ   ............................................................
HFC2B_ORYSJ   ............................................................
HFB2A_ORYSJ   EGADEDGEDDEVEEDDEERERHAARR...VPVREGKVRRTTELSDLDVLALSVR..AAAA
HFB4B_ORYSJ   ....LMELDSPDHSPPPPPPKTPATDGG...D..T.....VKLFGVSL...H......GR
HFB4D_ORYSJ   K.RRVQLVQEDEGDEQGSEG........................................
HFB2B_ORYSJ   .GGDD..DHA.ATVKAEPMDGRPHG.....KDEQSAETQAWPIYRPRPVYQPIR.ACNGY
HFB2C_ORYSJ   .GGEDQAEHGGADVKPEAADPHPAGGGGGSSTEASPESHPWPIYRPTPMYHAVRPTCNGP
HSFB1_ORYSJ   FGVLLDDTHGAATRKRARCEEAAASE...RPIKMIRIGEPW.........VSVP..SSGP
HFB4C_ORYSJ   ...P......PPQQQHLAGEKSGGEAGN...SSAARSSAPTKLFGVHLSAAP....CGAG
HFB4A_ORYSJ   ...TLLALRLPRTTEKIINEVSGGNGGG...S........TKLFGVHLSSADEQ.TSSGA

                   440       450       460       470       480
HFA1D_ARATH   V.P...DNMDSTPVDNETEQEQNGWDKTKHMDNLTQQMGLLSPETLDLSRQNP....
HSFA1_ORYSJ   L.SVDNDEVDSGVLDQKETQQGNGWTKAENMANLTEQMGLLS......SHHTG....
HFA2B_ORYSJ   .........................................................
HFA2E_ORYSJ   .........................................................
HFA2D_ORYSJ   .........................................................
HFA2C_ORYSJ   .........................................................
HFA2A_ORYSJ   .........................................................
HSFA9_ORYSJ   L.SV..QDYDFPQLEQDCLME......AQYNSNNPQYADVITEA.............
HSFA3_ORYSJ   QQAYGSLVSDPYLMEMANKPEKFWELDFQALDDGDLQLDKCVIDDPALQQQRGNMNS
HSFA5_ORYSJ   G.CSETEEASSGLRTDTSREQMENRQAYDHSRNDREDVEQLKL..............
HFA4B_ORYSJ   GSSDAHQEAQSERRDGGNKVDEMKSGDRQHLWWGKRNVEQITEKLGLLTSTEKT...
HFA6A_ORYSJ   .........................................................
HFA6B_ORYSJ   .........................................................
HFA4D_ORYSJ   RSCLDESERQESPKDDVKA..ELGCNGFHH....REKVDQITEQMGHLASAEQTLHT
HFC1A_ORYSJ   .........................................................
HFC1B_ORYSJ   .........................................................
HFC2A_ORYSJ   .........................................................
HFC2B_ORYSJ   .........................................................
HFB2A_ORYSJ   A..RPGGASRDRKSSVS........................................
HFB4B_ORYSJ   K.KRAHRDDDDGVHDQGSEV.....................................
HFB4D_ORYSJ   .........................................................
HFB2B_ORYSJ   EYDRAGSDQDGSNST..........................................
HFB2C_ORYSJ   ..DRAGSDQDGSSSSQTMGPGEF..DDLQKMMVVQQSNFVMHWGRSECGSGVRGFGW
HSFB1_ORYSJ   A..RCGGDN................................................
HFB4C_ORYSJ   S.KRASSPEEHPPTSPATKPRLV..LECDDLSLTVAPSSSSQQQLSAASSPTSTS..
HFB4A_ORYSJ   S.RKRSPPQEQPPTSPAPKRTLV..VEHSEL............RLSIVSPP......
```

Figure S13. Sequence alignment of Heat shock proteins of *Oryza sativa* against *Arabidospsis thaliana* HsFA1d. Multiple sequence alignment was constructed using full length sequences using BLAST. The alignment was depicted using Espript v.3. The AHA motif is highlighted in light pink. DNA binding domain (grey), HR-A region (blue), HR-B region (orange) and HR-A/B domain (pink) are indicated on the top of the alignment by continuous lines. The position of R181 in Arabidopsis HsFA1d is indicated with a pink arrow.
